# Relating a calcium indicator signal to the unperturbed calcium concentration time-course

**DOI:** 10.1186/1742-4682-4-7

**Published:** 2007-02-06

**Authors:** Alexander Borst, Henry DI Abarbanel

**Affiliations:** 1 Max-Planck-Institute of Neurobiology, Martinsried, Germany; 2 Department of Physics and Marine Physical Laboratory (Scripps Institution of Oceanography), University of California, San Diego, USA

## Abstract

**Background:**

Optical indicators of cytosolic calcium levels have become important experimental tools in systems and cellular neuroscience. Indicators are known to interfere with intracellular calcium levels by acting as additional buffers, and this may strongly alter the time-course of various dynamical variables to be measured.

**Results:**

By investigating the underlying reaction kinetics, we show that in some ranges of kinetic parameters one can explicitly link the time dependent indicator signal to the time-course of the calcium influx, and thus, to the unperturbed calcium level had there been no indicator in the cell.

## Background

The use of a fluorescent calcium indicator is a familiar technique for detecting dynamical changes in intracellular calcium levels [1-8]. However, introduction of the indicator into the cytosol inevitably perturbs the time-course of free cytosolic calcium by acting as a buffer, thus altering the quantity to be measured. To address this, traditional approaches to quantifying free cytosolic calcium have often restricted the use of the indicators to minimal concentrations with minimal affinity. While this minimizes the perturbation of the free calcium signal, it leads to the problem of small signal-to-noise ratios.

As an alternative approach, we examine here the dynamical equations for this process in various parameter ranges in order to identify the conditions under which approximate solutions can be obtained, allowing calcium influx to be calculated directly from the fluorescence time course measurements. Knowing the calcium influx, the free cytosolic calcium can then be calculated as if there had been no indicator in the cytosol.

In the following, we will denote the temporal derivative of a variable dx(t)/dt by the symbol x'(t). Furthermore, we will use the following symbols with the units shown in Table 1.

**Table 1:** Symbols used in kinetic model

**Symbol**	**Name of Quantity**	**Units**
*V*(*t*)	Membrane voltage	mV
x(t)	Free calcium concentration	Mol
y(t)	Indicator bound calcium	Mol
y_max_	Total free and bound indicator	Mol
*α*(t)	Calcium influx	Mol/sec
*γ*	Pump rate	1/sec
k_f_	Forward binding constant	1/(Mol sec)
k_b_	Backward binding constant	1/sec
K_D_	Dissociation constant = k_b_/k_f_	Mol
R_f_	dimensionless forward rate k_f_y_max_/*γ*	-
R_b_	dimensionless backward rate k_b_/γ	-

## Results

Upon activation of a neuron, calcium influx *α*(*t*) leads to an increase of the cytosolic calcium concentration. Once inside the cell, calcium goes one of two ways: either it is cleared from the cell, in proportion to its concentration, at a rate *γ*, or it binds to an indicator with forward and backward binding rates k_f _and k_b_, respectively. Processes such as diffusion, internal buffering, and release from internal calcium stores or extrusion by calcium-sodium exchangers are not considered here. We call the free calcium concentration x(t) with an intracellular calcium indicator, and the concentration of indicator with bound calcium y(t). We call the total indicator concentration (free and calcium bound) y_max_. Thus, the free indicator concentration becomes y_max_-y(t). The concentration y_max _is the initial level of the free indicator dye immediately after injection in the case of synthetic dyes, or the total amount of indicator protein (calcium bound and free) in the case of genetically encoded indicators.

The following system of two coupled nonlinear ordinary differential equations describes the dynamics of the system:

(1)     *x*'(*t*) = *α*(*t*) - *γ*·*x*(*t*) - *y*'(*t*)

(2)     *y*'(*t*) = *k*_*f*_·*x*(*t*)·[*y*_max _- *y*(*t*)] - *k*_*b*_·*y*(*t*)

The first equation state that the rate of change of free calcium, x'(t), is driven by the calcium flux *α*(*t*) and depleted by the pump -*γx*(*t*) as well as the rate of change of indicator bound to calcium, -y'(t). The second equation states that calcium is bound to the indicator at a rate k_f _and is proportional to the concentration of free calcium, x(t), as well as to the concentration of the free indicator, y_max _- y(t). Calcium disassociates from the indicator at a rate k_b _and this dissociation process is proportional to the concentration of calcium bound indicator y(t).

For a constant calcium influx *α*(t) = *α*_C_, the steady-state solutions are

(3)     *x*_∞ _= *α*_*C*_/*γ*, and

(4)     y∞=ymax⁡⋅αCαC+KD⋅γ
 MathType@MTEF@5@5@+=feaafiart1ev1aaatCvAUfKttLearuWrP9MDH5MBPbIqV92AaeXatLxBI9gBaebbnrfifHhDYfgasaacH8akY=wiFfYdH8Gipec8Eeeu0xXdbba9frFj0=OqFfea0dXdd9vqai=hGuQ8kuc9pgc9s8qqaq=dirpe0xb9q8qiLsFr0=vr0=vr0dc8meaabaqaciaacaGaaeqabaqabeGadaaakeaacqWG5bqEdaWgaaWcbaGaeyOhIukabeaakiabg2da9iabdMha5naaBaaaleaacyGGTbqBcqGGHbqycqGG4baEaeqaaOGaeyyXIC9aaSaaaeaaiiGacqWFXoqydaWgaaWcbaGaem4qameabeaaaOqaaiab=f7aHnaaBaaaleaacqWGdbWqaeqaaOGaey4kaSIaem4saS0aaSbaaSqaaiabdseaebqabaGccqGHflY1cqWFZoWzaaaaaa@4603@,     or in terms of x:     y∞=ymax⁡⋅x∞x∞+KD
 MathType@MTEF@5@5@+=feaafiart1ev1aaatCvAUfKttLearuWrP9MDH5MBPbIqV92AaeXatLxBI9gBaebbnrfifHhDYfgasaacH8akY=wiFfYdH8Gipec8Eeeu0xXdbba9frFj0=OqFfea0dXdd9vqai=hGuQ8kuc9pgc9s8qqaq=dirpe0xb9q8qiLsFr0=vr0=vr0dc8meaabaqaciaacaGaaeqabaqabeGadaaakeaacqWG5bqEdaWgaaWcbaGaeyOhIukabeaakiabg2da9iabdMha5naaBaaaleaacyGGTbqBcqGGHbqycqGG4baEaeqaaOGaeyyXIC9aaSaaaeaacqWG4baEdaWgaaWcbaGaeyOhIukabeaaaOqaaiabdIha4naaBaaaleaacqGHEisPaeqaaOGaey4kaSIaem4saS0aaSbaaSqaaiabdseaebqabaaaaaaa@4283@.

In general, eqs. (1) and (2) can only be solved numerically. However, if the indicator concentration is negligible compared to the calcium concentrations, eq. (1) turns into a simple differential equation describing a 1^st ^order low-pass filter with time-constant 1/*γ*:

(5)     *x*'(*t*) = *α*(*t*) - *γ*·*x*(*t*)

In other words: if there is no indicator present, and the pump rate is known, the unperturbed calcium concentration can be calculated as the low-pass filtered response to the calcium influx *α*(*t*).

Our approach will be to use eqs. (1) and (2) to determine the calcium influx *α*(*t*) in the presence of the indicator. This tells us how much calcium flows into the neuron as a result of activation, and allows us to remove the action of the indicator mathematically. With *α*(*t*) known, we may use eq. (5) to determine the time-course of the unperturbed calcium concentration. As we will show in the following, this approach is feasible only within certain parameter regimes, but is not restricted to the linear regime. Nevertheless, we will start our considerations with an analysis of the linear regime.

### The linear regime

To investigate the linear regime, we rewrite eq. (2) as

(6)     *y*'(*t*)/*k*_*f *_= *y*_max_·*x*(*t*) - *y*(*t*)·[*x*(*t*) - *K*_*D*_];

When *x*(*t*) is much smaller than the *K*_*D *_value of the indicator, eq. (6) becomes:

(7)     *y*'(*t*) = *y*_max_·*k*_*f*_·*x*(*t*) - *k*_*b*_·*y*(*t*)

Combining the derivative of this with eq. (1) gives us

(8)     *y*''(*t*) + *y*'(*t*)·(*k*_*b *_+ *γ *+ *k*_*f*_*y*_max_) + *y*(*t*)·*γ*·*k*_*b *_- *α*(*t*)·*k*_*f*_·*y*_max _= 0

This is a linear ordinary differential equation with constant coefficients. The solution of the homogeneous equation is of the form *y*(*t*) = *c*·*e*^*λ*·*t*^, where *λ *satisfies the characteristic equation:

(9)     *λ*^2 ^+ *λA *+ *γ*·*k*_*b *_= 0;     *A *= *k*_*b *_+ *γ *+ *k*_*f*_*y*_max_.

This has solutions *λ*_1,2 _with the negative inverses *τ*_1,2 _= -1/*λ*_1,2_, which are time-constants given by

(10)τ1,2=12γkb[kb+γ+kfymax⁡±(kb+γ+kfymax⁡)2−4γkb]
 MathType@MTEF@5@5@+=feaafiart1ev1aaatCvAUfKttLearuWrP9MDH5MBPbIqV92AaeXatLxBI9gBaebbnrfifHhDYfgasaacH8akY=wiFfYdH8Gipec8Eeeu0xXdbba9frFj0=OqFfea0dXdd9vqai=hGuQ8kuc9pgc9s8qqaq=dirpe0xb9q8qiLsFr0=vr0=vr0dc8meaabaqaciaacaGaaeqabaqabeGadaaakeaafaqabeqacaaabaWaaeWaaeaacqaIXaqmcqaIWaamaiaawIcacaGLPaaaaeaaiiGacqWFepaDdaWgaaWcbaGaeGymaeJaeiilaWIaeGOmaidabeaakiabg2da9maalaaabaGaeGymaedabaGaeGOmaiJae83SdCMaem4AaS2aaSbaaSqaaiabdkgaIbqabaaaaOWaamWaaeaacqWGRbWAdaWgaaWcbaGaemOyaigabeaakiabgUcaRiab=n7aNjabgUcaRiabdUgaRnaaBaaaleaacqWGMbGzaeqaaOGaemyEaK3aaSbaaSqaaiGbc2gaTjabcggaHjabcIha4bqabaGccqGHXcqSdaGcaaqaamaabmaabaGaem4AaS2aaSbaaSqaaiabdkgaIbqabaGccqGHRaWkcqWFZoWzcqGHRaWkcqWGRbWAdaWgaaWcbaGaemOzaygabeaakiabdMha5naaBaaaleaacyGGTbqBcqGGHbqycqGG4baEaeqaaaGccaGLOaGaayzkaaWaaWbaaSqabeaacqaIYaGmaaGccqGHsislcqaI0aancqWFZoWzcqWGRbWAdaWgaaWcbaGaemOyaigabeaaaeqaaaGccaGLBbGaayzxaaaaaaaa@677B@

Since (*k*_*b *_+ *γ *+ *k*_*f*_*y*_max_)^2 ^- 4*γ*·*k*_*b *_≥ 0 and kb+γ+kfymax⁡≥(kb+γ+kfymax⁡)2−4γ⋅kb
 MathType@MTEF@5@5@+=feaafiart1ev1aaatCvAUfKttLearuWrP9MDH5MBPbIqV92AaeXatLxBI9gBaebbnrfifHhDYfgasaacH8akY=wiFfYdH8Gipec8Eeeu0xXdbba9frFj0=OqFfea0dXdd9vqai=hGuQ8kuc9pgc9s8qqaq=dirpe0xb9q8qiLsFr0=vr0=vr0dc8meaabaqaciaacaGaaeqabaqabeGadaaakeaacqWGRbWAdaWgaaWcbaGaemOyaigabeaakiabgUcaRGGaciab=n7aNjabgUcaRiabdUgaRnaaBaaaleaacqWGMbGzaeqaaOGaemyEaK3aaSbaaSqaaiGbc2gaTjabcggaHjabcIha4bqabaGccqGHLjYSdaGcaaqaamaabmaabaGaem4AaS2aaSbaaSqaaiabdkgaIbqabaGccqGHRaWkcqWFZoWzcqGHRaWkcqWGRbWAdaWgaaWcbaGaemOzaygabeaakiabdMha5naaBaaaleaacyGGTbqBcqGGHbqycqGG4baEaeqaaaGccaGLOaGaayzkaaWaaWbaaSqabeaacqaIYaGmaaGccqGHsislcqaI0aancqWFZoWzcqGHflY1cqWGRbWAdaWgaaWcbaGaemOyaigabeaaaeqaaaaa@57F9@, both time-constants are always real and positive. For small values of k_f _y_max_, as well as for large values of k_b_, these become:

(11)lim⁡kfymax⁡→0τ1/2=lim⁡kb→∞τ1/2=1/γ.
 MathType@MTEF@5@5@+=feaafiart1ev1aaatCvAUfKttLearuWrP9MDH5MBPbIqV92AaeXatLxBI9gBaebbnrfifHhDYfgasaacH8akY=wiFfYdH8Gipec8Eeeu0xXdbba9frFj0=OqFfea0dXdd9vqai=hGuQ8kuc9pgc9s8qqaq=dirpe0xb9q8qiLsFr0=vr0=vr0dc8meaabaqaciaacaGaaeqabaqabeGadaaakeaafaqabeqacaaabaWaaeWaaeaacqaIXaqmcqaIXaqmaiaawIcacaGLPaaaaeaadaWfqaqaaiGbcYgaSjabcMgaPjabc2gaTbWcbaGaem4AaS2aaSbaaWqaaiabdAgaMbqabaWccqWG5bqEdaWgaaadbaGagiyBa0MaeiyyaeMaeiiEaGhabeaaliabgkziUkabicdaWaqabaacciGccqWFepaDdaWgaaWcbaGaeGymaeJaei4la8IaeGOmaidabeaakiabg2da9maaxababaGagiiBaWMaeiyAaKMaeiyBa0galeaacqWGRbWAdaWgaaadbaGaemOyaigabeaaliabgkziUkabg6HiLcqabaGccqWFepaDdaWgaaWcbaGaeGymaeJaei4la8IaeGOmaidabeaakiabg2da9iabigdaXiabc+caViab=n7aNjabc6caUaaaaaa@5AB6@

**Figure 1:**
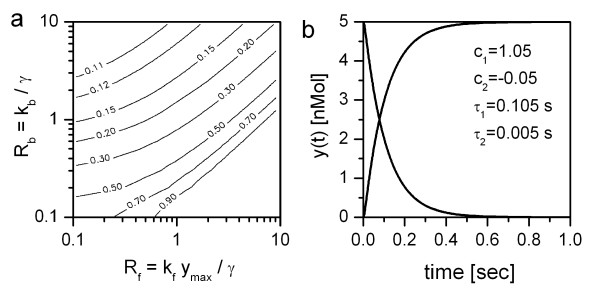
**a**: Dependence of the time-constant *τ*_1 _of the linearized system on the two dimensionless kinetic parameters R_b _and R_f_, shown as a contour plot in the R_b_-R_f _plane. Numbers on the iso-*τ *lines indicate the value of the time-constant in seconds. **b**: Time-course of the calcium-bound indicator signal y(t) in the linear regime, i.e. when eqs. (15) and (16) apply. The inset shows the parameters for the two exponential functions describing the time course: y(t)=y(0)⋅(c1⋅e−t/τ1+c2⋅e−t/τ2)
 MathType@MTEF@5@5@+=feaafiart1ev1aaatCvAUfKttLearuWrP9MDH5MBPbIqV92AaeXatLxBI9gBaebbnrfifHhDYfgasaacH8akY=wiFfYdH8Gipec8Eeeu0xXdbba9frFj0=OqFfea0dXdd9vqai=hGuQ8kuc9pgc9s8qqaq=dirpe0xb9q8qiLsFr0=vr0=vr0dc8meaabaqaciaacaGaaeqabaqabeGadaaakeaacqWG5bqEcqGGOaakcqWG0baDcqGGPaqkcqGH9aqpcqWG5bqEcqGGOaakcqaIWaamcqGGPaqkcqGHflY1daqadaqaaiabdogaJnaaBaaaleaacqaIXaqmaeqaaOGaeyyXICTaemyzau2aaWbaaSqabeaacqGHsislcqWG0baDcqGGVaWliiGacqWFepaDdaWgaaadbaGaeGymaedabeaaaaGccqGHRaWkcqWGJbWydaWgaaWcbaGaeGOmaidabeaakiabgwSixlabdwgaLnaaCaaaleqabaGaeyOeI0IaemiDaqNaei4la8Iae8hXdq3aaSbaaWqaaiabikdaYaqabaaaaaGccaGLOaGaayzkaaaaaa@5404@ for the decay, and y(t)=y(0)⋅(1−c1⋅e−t/τ1+c2⋅e−t/τ2)
 MathType@MTEF@5@5@+=feaafiart1ev1aaatCvAUfKttLearuWrP9MDH5MBPbIqV92AaeXatLxBI9gBaebbnrfifHhDYfgasaacH8akY=wiFfYdH8Gipec8Eeeu0xXdbba9frFj0=OqFfea0dXdd9vqai=hGuQ8kuc9pgc9s8qqaq=dirpe0xb9q8qiLsFr0=vr0=vr0dc8meaabaqaciaacaGaaeqabaqabeGadaaakeaacqWG5bqEcqGGOaakcqWG0baDcqGGPaqkcqGH9aqpcqWG5bqEcqGGOaakcqaIWaamcqGGPaqkcqGHflY1daqadaqaaiabigdaXiabgkHiTiabdogaJnaaBaaaleaacqaIXaqmaeqaaOGaeyyXICTaemyzau2aaWbaaSqabeaacqGHsislcqWG0baDcqGGVaWliiGacqWFepaDdaWgaaadbaGaeGymaedabeaaaaGccqGHRaWkcqWGJbWydaWgaaWcbaGaeGOmaidabeaakiabgwSixlabdwgaLnaaCaaaleqabaGaeyOeI0IaemiDaqNaei4la8Iae8hXdq3aaSbaaWqaaiabikdaYaqabaaaaaGccaGLOaGaayzkaaaaaa@55E1@ for the increase. If not subject to variation, the parameters in both a and b were as follows: k_b _= 100.0 [1/sec], k_f _= 0.1 [1/(nMol*sec)], *γ *= 20.0 [1/sec], y_max _= 1000.0 [nMol] and *α*_0 _= 100.0 [nMol/sec]. Consequently, the K_D _= 1000 nMol, and the dimensionless kinetic parameters R_b _and R_f _both were 5.0.

The dependence of the time-constant with the larger absolute value, *τ*_1_, on the dimensionless parameters R_b _and R_f_,

(12)τ1=12γRb[Rb+Rf+1+(Rb+Rf+1)2−4Rb]
 MathType@MTEF@5@5@+=feaafiart1ev1aaatCvAUfKttLearuWrP9MDH5MBPbIqV92AaeXatLxBI9gBaebbnrfifHhDYfgasaacH8akY=wiFfYdH8Gipec8Eeeu0xXdbba9frFj0=OqFfea0dXdd9vqai=hGuQ8kuc9pgc9s8qqaq=dirpe0xb9q8qiLsFr0=vr0=vr0dc8meaabaqaciaacaGaaeqabaqabeGadaaakeaafaqabeqacaaabaWaaeWaaeaacqaIXaqmcqaIYaGmaiaawIcacaGLPaaaaeaaiiGacqWFepaDdaWgaaWcbaGaeGymaedabeaakiabg2da9maalaaabaGaeGymaedabaGaeGOmaiJae83SdCMaemOuai1aaSbaaSqaaiabdkgaIbqabaaaaOWaamWaaeaacqWGsbGudaWgaaWcbaGaemOyaigabeaakiabgUcaRiabdkfasnaaBaaaleaacqWGMbGzaeqaaOGaey4kaSIaeGymaeJaey4kaSYaaOaaaeaadaqadaqaaiabdkfasnaaBaaaleaacqWGIbGyaeqaaOGaey4kaSIaemOuai1aaSbaaSqaaiabdAgaMbqabaGccqGHRaWkcqaIXaqmaiaawIcacaGLPaaadaahaaWcbeqaaiabikdaYaaakiabgkHiTiabisda0iabdkfasnaaBaaaleaacqWGIbGyaeqaaaqabaaakiaawUfacaGLDbaaaaaaaa@54C1@

is shown in Figure 1a. The axes are logarithmic. As one can see, the larger we set R_b_, at fixed R_f_, the smaller is *τ*_1_, that is, the faster calcium is released from the bound indicator. *τ*_1 _is larger, at fixed R_b_, for larger R_f_; in other words, the faster calcium is bound to the indicator (k_f_) and the larger the initial indicator concentration (y_max_).

For the case of a pulse of injected calcium current of sufficient length, we can obtain particular solutions for *y*(*t*). For that, we insert y(t)=c1⋅eλ1⋅t+c2⋅eλ2⋅t+k
 MathType@MTEF@5@5@+=feaafiart1ev1aaatCvAUfKttLearuWrP9MDH5MBPbIqV92AaeXatLxBI9gBaebbnrfifHhDYfgasaacH8akY=wiFfYdH8Gipec8Eeeu0xXdbba9frFj0=OqFfea0dXdd9vqai=hGuQ8kuc9pgc9s8qqaq=dirpe0xb9q8qiLsFr0=vr0=vr0dc8meaabaqaciaacaGaaeqabaqabeGadaaakeaacqWG5bqEcqGGOaakcqWG0baDcqGGPaqkcqGH9aqpcqWGJbWydaWgaaWcbaGaeGymaedabeaakiabgwSixlabdwgaLnaaCaaaleqabaacciGae83UdW2aaSbaaWqaaiabigdaXaqabaWccqGHflY1cqWG0baDaaGccqGHRaWkcqWGJbWydaWgaaWcbaGaeGOmaidabeaakiabgwSixlabdwgaLnaaCaaaleqabaGae83UdW2aaSbaaWqaaiabikdaYaqabaWccqGHflY1cqWG0baDaaGccqGHRaWkcqWGRbWAaaa@4F39@ into eq. (8) and note the following initial conditions: *y*(0) = 0 and *y*'(0) = 0 for the rise of *y*(*t*) after the pulse is initiated, and *y*(0) = *y*_max_*α*_*c*_/(*γ*·*K*_*D*_) and *y*'(0) = 0 for the decay phase after the pulse is completed.

The initial increase of bound indicator from *y*(*t*) = 0, using *A *= *k*_*b *_+ *γ *+ *k*_*f*_·*y*_max _again, is

(13)y(t)=ymax⁡⋅αC2⋅γ⋅KD⋅[1−(1+AA2−4kbγ)⋅exp⁡(−t/τ1)−(1−AA2−4kbγ)⋅exp⁡(−t/τ2)]
 MathType@MTEF@5@5@+=feaafiart1ev1aaatCvAUfKttLearuWrP9MDH5MBPbIqV92AaeXatLxBI9gBaebbnrfifHhDYfgasaacH8akY=wiFfYdH8Gipec8Eeeu0xXdbba9frFj0=OqFfea0dXdd9vqai=hGuQ8kuc9pgc9s8qqaq=dirpe0xb9q8qiLsFr0=vr0=vr0dc8meaabaqaciaacaGaaeqabaqabeGadaaakeaafaqabeqacaaabaWaaeWaaeaacqaIXaqmcqaIZaWmaiaawIcacaGLPaaaaeaacqWG5bqEcqGGOaakcqWG0baDcqGGPaqkcqGH9aqpdaWcaaqaaiabdMha5naaBaaaleaacyGGTbqBcqGGHbqycqGG4baEaeqaaOGaeyyXICncciGae8xSde2aaSbaaSqaaiabdoeadbqabaaakeaacqaIYaGmcqGHflY1cqWFZoWzcqGHflY1cqWGlbWsdaWgaaWcbaGaemiraqeabeaaaaGccqGHflY1daWadaqaaiabigdaXiabgkHiTmaabmaabaGaeGymaeJaey4kaSYaaSaaaeaacqWGbbqqaeaadaGcaaqaaiabdgeabnaaCaaaleqabaGaeGOmaidaaOGaeyOeI0IaeGinaqJaem4AaS2aaSbaaSqaaiabdkgaIbqabaGccqWFZoWzaSqabaaaaaGccaGLOaGaayzkaaGaeyyXICTagiyzauMaeiiEaGNaeiiCaa3aaeWaaeaacqGHsislcqWG0baDcqGGVaWlcqWFepaDdaWgaaWcbaGaeGymaedabeaaaOGaayjkaiaawMcaaiabgkHiTmaabmaabaGaeGymaeJaeyOeI0YaaSaaaeaacqWGbbqqaeaadaGcaaqaaiabdgeabnaaCaaaleqabaGaeGOmaidaaOGaeyOeI0IaeGinaqJaem4AaS2aaSbaaSqaaiabdkgaIbqabaGccqWFZoWzaSqabaaaaaGccaGLOaGaayzkaaGaeyyXICTagiyzauMaeiiEaGNaeiiCaa3aaeWaaeaacqGHsislcqWG0baDcqGGVaWlcqWFepaDdaWgaaWcbaGaeGOmaidabeaaaOGaayjkaiaawMcaaaGaay5waiaaw2faaaaaaaa@8855@

After calcium influx has stopped, when *α*(*t*) = 0, the bound indicator decays to zero as

(14)y(t)=ymax⁡⋅αC2⋅γ⋅KD⋅[(1+AA2−4kbγ)⋅exp⁡(−t/τ1)+(1−AA2−4kbγ)⋅exp⁡(−t/τ2)]
 MathType@MTEF@5@5@+=feaafiart1ev1aaatCvAUfKttLearuWrP9MDH5MBPbIqV92AaeXatLxBI9gBaebbnrfifHhDYfgasaacH8akY=wiFfYdH8Gipec8Eeeu0xXdbba9frFj0=OqFfea0dXdd9vqai=hGuQ8kuc9pgc9s8qqaq=dirpe0xb9q8qiLsFr0=vr0=vr0dc8meaabaqaciaacaGaaeqabaqabeGadaaakeaafaqabeqacaaabaWaaeWaaeaacqaIXaqmcqaI0aanaiaawIcacaGLPaaaaeaacqWG5bqEcqGGOaakcqWG0baDcqGGPaqkcqGH9aqpdaWcaaqaaiabdMha5naaBaaaleaacyGGTbqBcqGGHbqycqGG4baEaeqaaOGaeyyXICncciGae8xSde2aaSbaaSqaaiabdoeadbqabaaakeaacqaIYaGmcqGHflY1cqWFZoWzcqGHflY1cqWGlbWsdaWgaaWcbaGaemiraqeabeaaaaGccqGHflY1daWadaqaamaabmaabaGaeGymaeJaey4kaSYaaSaaaeaacqWGbbqqaeaadaGcaaqaaiabdgeabnaaCaaaleqabaGaeGOmaidaaOGaeyOeI0IaeGinaqJaem4AaS2aaSbaaSqaaiabdkgaIbqabaGccqWFZoWzaSqabaaaaaGccaGLOaGaayzkaaGaeyyXICTagiyzauMaeiiEaGNaeiiCaa3aaeWaaeaacqGHsislcqWG0baDcqGGVaWlcqWFepaDdaWgaaWcbaGaeGymaedabeaaaOGaayjkaiaawMcaaiabgUcaRmaabmaabaGaeGymaeJaeyOeI0YaaSaaaeaacqWGbbqqaeaadaGcaaqaaiabdgeabnaaCaaaleqabaGaeGOmaidaaOGaeyOeI0IaeGinaqJaem4AaS2aaSbaaSqaaiabdkgaIbqabaGccqWFZoWzaSqabaaaaaGccaGLOaGaayzkaaGaeyyXICTagiyzauMaeiiEaGNaeiiCaa3aaeWaaeaacqGHsislcqWG0baDcqGGVaWlcqWFepaDdaWgaaWcbaGaeGOmaidabeaaaOGaayjkaiaawMcaaaGaay5waiaaw2faaaaaaaa@866F@

The time course of the indicator signal under these conditions is shown in Fig. 1b.

The goal of this paper is to use the dynamical equations to determine the *α*(*t*) associated with an observed indicator signal *y*(*t*), and then relate that to the free calcium concentration that would be associated with this *α*(*t*) when the indicator is absent. In the linear regime under consideration, we need to solve eq. (8) for *α*(*t*) to obtain

(15)α(t)=1kfymax⁡[y″(t)+y′(t)(kb+γ+kfymax⁡)+y(t)⋅γ⋅kb]
 MathType@MTEF@5@5@+=feaafiart1ev1aaatCvAUfKttLearuWrP9MDH5MBPbIqV92AaeXatLxBI9gBaebbnrfifHhDYfgasaacH8akY=wiFfYdH8Gipec8Eeeu0xXdbba9frFj0=OqFfea0dXdd9vqai=hGuQ8kuc9pgc9s8qqaq=dirpe0xb9q8qiLsFr0=vr0=vr0dc8meaabaqaciaacaGaaeqabaqabeGadaaakeaafaqabeqacaaabaWaaeWaaeaacqaIXaqmcqaI1aqnaiaawIcacaGLPaaaaeaaiiGacqWFXoqycqGGOaakcqWG0baDcqGGPaqkcqGH9aqpdaWcaaqaaiabigdaXaqaaiabdUgaRnaaBaaaleaacqWGMbGzaeqaaOGaemyEaK3aaSbaaSqaaiGbc2gaTjabcggaHjabcIha4bqabaaaaOWaamWaaeaacuWG5bqEgaGbaiabcIcaOiabdsha0jabcMcaPiabgUcaRiqbdMha5zaafaGaeiikaGIaemiDaqNaeiykaKYaaeWaaeaacqWGRbWAdaWgaaWcbaGaemOyaigabeaakiabgUcaRiab=n7aNjabgUcaRiabdUgaRnaaBaaaleaacqWGMbGzaeqaaOGaemyEaK3aaSbaaSqaaiGbc2gaTjabcggaHjabcIha4bqabaaakiaawIcacaGLPaaacqGHRaWkcqWG5bqEcqGGOaakcqWG0baDcqGGPaqkcqGHflY1cqWFZoWzcqGHflY1cqWGRbWAdaWgaaWcbaGaemOyaigabeaaaOGaay5waiaaw2faaaaaaaa@6B0D@

From this *α*(*t*) the unperturbed time-course of the calcium concentration x*(t) can be calculated from (1). It is the response of a 1^st ^order low-pass filter with time-constant 1/*γ *to the driving input *α*(*t*):

(16)x∗(t)=∫0tdt′α(t−t′)e−γ⋅t′
MathType@MTEF@5@5@+=feaafiart1ev1aaatCvAUfKttLearuWrP9MDH5MBPbIqV92AaeXatLxBI9gBaebbnrfifHhDYfgasaacH8akY=wiFfYdH8Gipec8Eeeu0xXdbba9frFj0=OqFfea0dXdd9vqai=hGuQ8kuc9pgc9s8qqaq=dirpe0xb9q8qiLsFr0=vr0=vr0dc8meaabaqaciaacaGaaeqabaqabeGadaaakeaafaqabeqacaaabaWaaeWaaeaacqaIXaqmcqaI2aGnaiaawIcacaGLPaaaaeaacqWG4baEdaahaaWcbeqaaiabgEHiQaaakiabcIcaOiabdsha0jabcMcaPiabg2da9maapehabaGaemizaqMafmiDaqNbauaaiiGacqWFXoqycqGGOaakcqWG0baDcqGHsislcuWG0baDgaqbaiabcMcaPiabdwgaLnaaCaaaleqabaGaeyOeI0Iae83SdCMaeyyXICTafmiDaqNbauaaaaaabaGaeGimaadabaGaemiDaqhaniabgUIiYdaaaaaa@4D88@

From eq. (15) it also follows that lim⁡ymax⁡→∞α(t)=y′(t)
 MathType@MTEF@5@5@+=feaafiart1ev1aaatCvAUfKttLearuWrP9MDH5MBPbIqV92AaeXatLxBI9gBaebbnrfifHhDYfgasaacH8akY=wiFfYdH8Gipec8Eeeu0xXdbba9frFj0=OqFfea0dXdd9vqai=hGuQ8kuc9pgc9s8qqaq=dirpe0xb9q8qiLsFr0=vr0=vr0dc8meaabaqaciaacaGaaeqabaqabeGadaaakeaadaWfqaqaaiGbcYgaSjabcMgaPjabc2gaTbWcbaGaemyEaK3aaSbaaWqaaiGbc2gaTjabcggaHjabcIha4bqabaWccqGHsgIRcqGHEisPaeqaaGGacOGae8xSdeMaeiikaGIaemiDaqNaeiykaKIaeyypa0JafmyEaKNbauaacqGGOaakcqWG0baDcqGGPaqkaaa@44BD@. This is expected as infinite indicator promptly binds all the available free calcium. So when the cell is overloaded, the indicator signal directly integrates the calcium influx: the influx can conversely be recovered by simply differentiating the indicator signal. This completes our discussion of the linear regime and we turn to the nonlinear equations again.

### Approximate solution in the nonlinear regime

If we examine the nonlinear eqs. (1) and (2) we see that an approximate solution with small rate of change in the calcium bound to the indicator y'(t) is given by

(17)y(t)=ymax⁡⋅x(t)x(t)+KD.
 MathType@MTEF@5@5@+=feaafiart1ev1aaatCvAUfKttLearuWrP9MDH5MBPbIqV92AaeXatLxBI9gBaebbnrfifHhDYfgasaacH8akY=wiFfYdH8Gipec8Eeeu0xXdbba9frFj0=OqFfea0dXdd9vqai=hGuQ8kuc9pgc9s8qqaq=dirpe0xb9q8qiLsFr0=vr0=vr0dc8meaabaqaciaacaGaaeqabaqabeGadaaakeaafaqabeqacaaabaWaaeWaaeaacqaIXaqmcqaI3aWnaiaawIcacaGLPaaaaeaacqWG5bqEcqGGOaakcqWG0baDcqGGPaqkcqGH9aqpdaWcaaqaaiabdMha5naaBaaaleaacyGGTbqBcqGGHbqycqGG4baEaeqaaOGaeyyXICTaemiEaGNaeiikaGIaemiDaqNaeiykaKcabaGaemiEaGNaeiikaGIaemiDaqNaeiykaKIaey4kaSIaem4saS0aaSbaaSqaaiabdseaebqabaaaaOGaeiOla4caaaaa@4B67@

This is an exact solution when *α*(*t*) is constant, and *x*(*t*) and *y*(*t*) are at the fixed point discussed earlier. So, this might well be a good guess for an approximate solution of the overall equations. We discuss this in the appendix, and argue that as long as *x*(*t*) is bounded, perturbations to this solution decay back to it at a rate to be established there. Also, the variations in *x*(*t*) are required to be slow compared to the variations in the perturbations. This means the frequency of the low pass filter giving *x*(*t*) from the calcium flux should be smaller than the decay frequencies of the perturbation. The time constant for the low-pass filter is 1/*γ*.

If we use this solution, i.e. eq. (17), we have

(18)x(t)=y(t)KDymax⁡−y(t)andx′(t)=y′(t)KDymax⁡(ymax⁡−y(t))2.
 MathType@MTEF@5@5@+=feaafiart1ev1aaatCvAUfKttLearuWrP9MDH5MBPbIqV92AaeXatLxBI9gBaebbnrfifHhDYfgasaacH8akY=wiFfYdH8Gipec8Eeeu0xXdbba9frFj0=OqFfea0dXdd9vqai=hGuQ8kuc9pgc9s8qqaq=dirpe0xb9q8qiLsFr0=vr0=vr0dc8meaabaqaciaacaGaaeqabaqabeGadaaakeaafaqabeqaeaaaaeaadaqadaqaaiabigdaXiabiIda4aGaayjkaiaawMcaaaqaaiabdIha4jabcIcaOiabdsha0jabcMcaPiabg2da9maalaaabaGaemyEaKNaeiikaGIaemiDaqNaeiykaKIaem4saS0aaSbaaSqaaiabdseaebqabaaakeaacqWG5bqEdaWgaaWcbaGagiyBa0MaeiyyaeMaeiiEaGhabeaakiabgkHiTiabdMha5jabcIcaOiabdsha0jabcMcaPaaaaeaacqqGHbqycqqGUbGBcqqGKbazaeaacuWG4baEgaqbaiabcIcaOiabdsha0jabcMcaPiabg2da9maalaaabaGafmyEaKNbauaacqGGOaakcqWG0baDcqGGPaqkcqWGlbWsdaWgaaWcbaGaemiraqeabeaakiabdMha5naaBaaaleaacyGGTbqBcqGGHbqycqGG4baEaeqaaaGcbaWaaeWaaeaacqWG5bqEdaWgaaWcbaGagiyBa0MaeiyyaeMaeiiEaGhabeaakiabgkHiTiabdMha5jabcIcaOiabdsha0jabcMcaPaGaayjkaiaawMcaamaaCaaaleqabaGaeGOmaidaaaaakiabc6caUaaaaaa@6DE4@

Substituting these terms in eq (1), we determine *α*(*t*) from the observed values of y(t) and y'(t):

(19)α(t)=γ⋅KD⋅y(t)ymax⁡−y(t)+y′(t)⋅[1+KDymax⁡(ymax⁡−y(t))2],
 MathType@MTEF@5@5@+=feaafiart1ev1aaatCvAUfKttLearuWrP9MDH5MBPbIqV92AaeXatLxBI9gBaebbnrfifHhDYfgasaacH8akY=wiFfYdH8Gipec8Eeeu0xXdbba9frFj0=OqFfea0dXdd9vqai=hGuQ8kuc9pgc9s8qqaq=dirpe0xb9q8qiLsFr0=vr0=vr0dc8meaabaqaciaacaGaaeqabaqabeGadaaakeaafaqabeqacaaabaWaaeWaaeaacqaIXaqmcqaI5aqoaiaawIcacaGLPaaaaeaaiiGacqWFXoqycqGGOaakcqWG0baDcqGGPaqkcqGH9aqpdaWcaaqaaiab=n7aNjabgwSixlabdUealnaaBaaaleaacqWGebaraeqaaOGaeyyXICTaemyEaKNaeiikaGIaemiDaqNaeiykaKcabaGaemyEaK3aaSbaaSqaaiGbc2gaTjabcggaHjabcIha4bqabaGccqGHsislcqWG5bqEcqGGOaakcqWG0baDcqGGPaqkaaGaey4kaSIafmyEaKNbauaacqGGOaakcqWG0baDcqGGPaqkcqGHflY1daWadaqaaiabigdaXiabgUcaRmaalaaabaGaem4saS0aaSbaaSqaaiabdseaebqabaGccqWG5bqEdaWgaaWcbaGagiyBa0MaeiyyaeMaeiiEaGhabeaaaOqaamaabmaabaGaemyEaK3aaSbaaSqaaiGbc2gaTjabcggaHjabcIha4bqabaGccqGHsislcqWG5bqEcqGGOaakcqWG0baDcqGGPaqkaiaawIcacaGLPaaadaahaaWcbeqaaiabikdaYaaaaaaakiaawUfacaGLDbaacqGGSaalaaaaaa@7188@

once again allowing us to determine the effective calcium flux from observations of the indicator signal, related to *y*(*t*) as discussed below. The time course of the equivalent unperturbed calcium signal is determined as in eq. (16). Note again that lim⁡ymax⁡→∞α(t)=y′(t)
 MathType@MTEF@5@5@+=feaafiart1ev1aaatCvAUfKttLearuWrP9MDH5MBPbIqV92AaeXatLxBI9gBaebbnrfifHhDYfgasaacH8akY=wiFfYdH8Gipec8Eeeu0xXdbba9frFj0=OqFfea0dXdd9vqai=hGuQ8kuc9pgc9s8qqaq=dirpe0xb9q8qiLsFr0=vr0=vr0dc8meaabaqaciaacaGaaeqabaqabeGadaaakeaadaWfqaqaaiGbcYgaSjabcMgaPjabc2gaTbWcbaGaemyEaK3aaSbaaWqaaiGbc2gaTjabcggaHjabcIha4bqabaWccqGHsgIRcqGHEisPaeqaaGGacOGae8xSdeMaeiikaGIaemiDaqNaeiykaKIaeyypa0JafmyEaKNbauaacqGGOaakcqWG0baDcqGGPaqkaaa@44BD@.

The critical question, of course, is under what circumstances this approximation is good. This requires the perturbation analysis in the appendix where we give the decay time constants (in dimensionless units) for small perturbations from the assumed solution (eq. (17)):

λ1,2=−C±D,C=12(1+Rb+RfX0+RbRfRb+RfX0),D=C2−(Rb+RfX0)<C.
 MathType@MTEF@5@5@+=feaafiart1ev1aaatCvAUfKttLearuWrP9MDH5MBPbIqV92AaeXatLxBI9gBaebbnrfifHhDYfgasaacH8akY=wiFfYdH8Gipec8Eeeu0xXdbba9frFj0=OqFfea0dXdd9vqai=hGuQ8kuc9pgc9s8qqaq=dirpe0xb9q8qiLsFr0=vr0=vr0dc8meaabaqaciaacaGaaeqabaqabeGadaaakeaafaqaaeWabaaabaacciGae83UdW2aaSbaaSqaaiabigdaXiabcYcaSiabikdaYaqabaGccqGH9aqpcqGHsislcqWGdbWqcqGHXcqScqWGebarcqGGSaalaeaacqWGdbWqcqGH9aqpdaWcaaqaaiabigdaXaqaaiabikdaYaaadaqadaqaaiabigdaXiabgUcaRiabdkfasnaaBaaaleaacqWGIbGyaeqaaOGaey4kaSIaemOuai1aaSbaaSqaaiabdAgaMbqabaGccqWGybawdaWgaaWcbaGaeGimaadabeaakiabgUcaRmaalaaabaGaemOuai1aaSbaaSqaaiabdkgaIbqabaGccqWGsbGudaWgaaWcbaGaemOzaygabeaaaOqaaiabdkfasnaaBaaaleaacqWGIbGyaeqaaOGaey4kaSIaemOuai1aaSbaaSqaaiabdAgaMbqabaGccqWGybawdaWgaaWcbaGaeGimaadabeaaaaaakiaawIcacaGLPaaacqGGSaalaeaacqWGebarcqGH9aqpdaGcaaqaaiabdoeadnaaCaaaleqabaGaeGOmaidaaOGaeyOeI0YaaeWaaeaacqWGsbGudaWgaaWcbaGaemOyaigabeaakiabgUcaRiabdkfasnaaBaaaleaacqWGMbGzaeqaaOGaemiwaG1aaSbaaSqaaiabicdaWaqabaaakiaawIcacaGLPaaaaSqabaGccqGH8aapcqWGdbWqcqGGUaGlaaaaaa@6AC0@

Here, X_0 _is a positive constant. Both time constants are negative, indicating decay of a perturbation back to the assumed solution. These inverse time constants, in dimensional form, must be greater than the low pass filter time constant 1/*γ *for the free calcium concentration. This is true in the regime of large dimensionless forward and backward rates.

**Figure 2:**
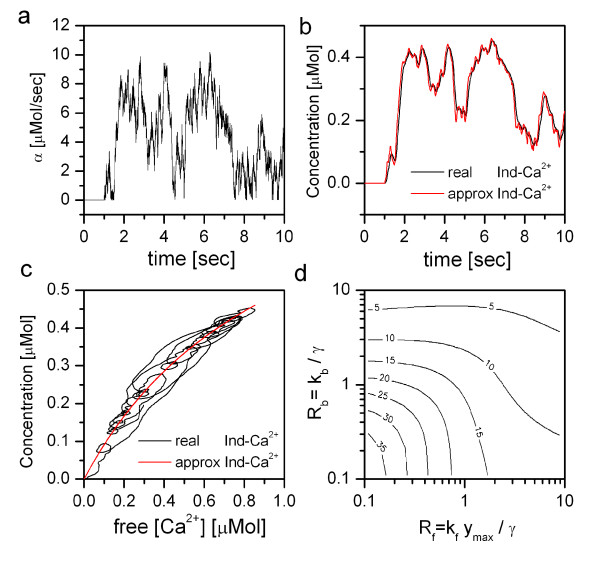
Results of numerical integration of eqs. (1) and (2). a: Calcium influx *α*(t). b: Real and approximated indicator-bound calcium concentrations, given the following parameters: pump rate *γ*(t) = 10 Hz, initial free indicator concentration y_max _= 1 *μ*Mol, indicator backward rate k_b _= 10 Hz and indicator forward rate k_f _= 10 Hz/*μ*Mol. This corresponds to Rb and Rf = 1.0. c: Real and approximated indicator-bound calcium as a function of the free cytosolic calcium. d: Root-mean-square (rms) of the difference between real and approximated signal as a function of the two dimensionless kinetic parameters Rb and Rf. Number on the iso-rms contour lines indicate the rms value as a percent of the real indicator-bound signal.

A numerical evaluation of the system of differential equations (eqs. (1) and (2)) is shown in Fig. 2. As calcium influx *α*(t) we used a white-noise signal with a standard deviation of 5 *μ*Mol/sec that was subsequently filtered by a 1^st^-order low-pass with 1 sec time-constant and finally rectified (Fig. 2a). This signal was then fed into eqs. (1) and (2), using the following parameters: pump rate *γ *= 10 Hz, initial free indicator concentration y_max _= 1 *μ*Mol, indicator backward rate k_b _= 10 Hz and indicator forward rate k_f _= 10 Hz/*μ*Mol. With these parameters, the resulting time-course of the indicator-bound calcium is shown in Fig. 2b. As a comparison, we also show in Fig. 2b the indicator-bound calcium approximated by eq. (17). Both curves closely agree. In Fig. 2c, the indicator-bound calcium is shown as a function of the free cytosolic calcium, once (in black) as obtained from numerical integration of eqs. (1) and (2), once (in red) using the approximation using eq. (17). In this plot, certain deviations of the real signal from the approximate one can be observed. We subsequently quantified these deviations by calculating the root-mean-square of the difference between the real and approximated signals. We did that for a total of 10,000 pairs of the two kinetic parameters R_b _and R_f _as defined above. Note that the parameters used in the above examples correspond to the values R_b _= 1.0 and R_f _= 1.0. The result is shown in Fig. 2d. The contour plot indicates that the rms values are smaller, i.e. the approximation is better, for larger R_b _and R_f _values. This is in close agreement with the result of our perturbation analysis.

### Including internal buffering

Our mathematical analysis, for the sake of simplicity, has so far excluded the existence of internal buffers. In the following, we introduce an additional variable z(t), denoting the calcium bound internal buffer. We also give a superscript to the rate constants with 'y' referring to the calcium bound indicator, and 'z' referring to the calcium bound internal buffer. Consequently, we call the total (free and calcium bound) buffer concentration z_max_. Writing down the basic dynamic equations gives:

(21)     *x*'(*t*) = *α*(*t*) - *γ*·*x*(*t*) - *y*'(*t*) - *z*'(*t*)

(22)y(t)=kfy⋅x(t)⋅[ymax⁡−y(t)]−kby⋅y(t)
 MathType@MTEF@5@5@+=feaafiart1ev1aaatCvAUfKttLearuWrP9MDH5MBPbIqV92AaeXatLxBI9gBaebbnrfifHhDYfgasaacH8akY=wiFfYdH8Gipec8Eeeu0xXdbba9frFj0=OqFfea0dXdd9vqai=hGuQ8kuc9pgc9s8qqaq=dirpe0xb9q8qiLsFr0=vr0=vr0dc8meaabaqaciaacaGaaeqabaqabeGadaaakeaafaqabeqacaaabaWaaeWaaeaacqaIYaGmcqaIYaGmaiaawIcacaGLPaaaaeaacqWG5bqEcqGGOaakcqWG0baDcqGGPaqkcqGH9aqpcqWGRbWAdaqhaaWcbaGaemOzaygabaGaemyEaKhaaOGaeyyXICTaemiEaGNaeiikaGIaemiDaqNaeiykaKIaeyyXIC9aamWaaeaacqWG5bqEdaWgaaWcbaGagiyBa0MaeiyyaeMaeiiEaGhabeaakiabgkHiTiabdMha5jabcIcaOiabdsha0jabcMcaPaGaay5waiaaw2faaaaacqGHsislcqWGRbWAdaqhaaWcbaGaemOyaigabaGaemyEaKhaaOGaeyyXICTaemyEaKNaeiikaGIaemiDaqNaeiykaKcaaa@5CE7@

(23)z′(t)=kfz⋅x(t)⋅[zmax⁡−z(t)]−kbz⋅z(t)
 MathType@MTEF@5@5@+=feaafiart1ev1aaatCvAUfKttLearuWrP9MDH5MBPbIqV92AaeXatLxBI9gBaebbnrfifHhDYfgasaacH8akY=wiFfYdH8Gipec8Eeeu0xXdbba9frFj0=OqFfea0dXdd9vqai=hGuQ8kuc9pgc9s8qqaq=dirpe0xb9q8qiLsFr0=vr0=vr0dc8meaabaqaciaacaGaaeqabaqabeGadaaakeaafaqabeqacaaabaWaaeWaaeaacqaIYaGmcqaIZaWmaiaawIcacaGLPaaaaeaacuWG6bGEgaqbaiabcIcaOiabdsha0jabcMcaPiabg2da9iabdUgaRnaaDaaaleaacqWGMbGzaeaacqWG6bGEaaGccqGHflY1cqWG4baEcqGGOaakcqWG0baDcqGGPaqkcqGHflY1daWadaqaaiabdQha6naaBaaaleaacyGGTbqBcqGGHbqycqGG4baEaeqaaOGaeyOeI0IaemOEaONaeiikaGIaemiDaqNaeiykaKcacaGLBbGaayzxaaGaeyOeI0Iaem4AaS2aa0baaSqaaiabdkgaIbqaaiabdQha6baakiabgwSixlabdQha6jabcIcaOiabdsha0jabcMcaPaaaaaa@5D01@

Comparing these equations with our initial set (eqs. (1) and (2)), one realizes that an additional loss term has entered in eq. (21) to account for the calcium binding to the internal buffer. Eq. (22), which describes the binding to the indicator, is identical to eq. (2), and eq. (23) is a replication of eq. (2) with the buffer z substituting for the indicator y.

The steady-state solutions are:

(24)x∞=αC/γ;y∞=ymax⁡⋅αCαC+KDy⋅γ;z∞=zmax⁡⋅αCαC+KDz⋅γ;
 MathType@MTEF@5@5@+=feaafiart1ev1aaatCvAUfKttLearuWrP9MDH5MBPbIqV92AaeXatLxBI9gBaebbnrfifHhDYfgasaacH8akY=wiFfYdH8Gipec8Eeeu0xXdbba9frFj0=OqFfea0dXdd9vqai=hGuQ8kuc9pgc9s8qqaq=dirpe0xb9q8qiLsFr0=vr0=vr0dc8meaabaqaciaacaGaaeqabaqabeGadaaakeaafaqabeqaeaaaaeaadaqadaqaaiabikdaYiabisda0aGaayjkaiaawMcaaaqaaiabdIha4naaBaaaleaacqGHEisPaeqaaOGaeyypa0dcciGae8xSde2aaSbaaSqaaiabdoeadbqabaGccqGGVaWlcqWFZoWzcqGG7aWoaeaacqWG5bqEdaWgaaWcbaGaeyOhIukabeaakiabg2da9iabdMha5naaBaaaleaacyGGTbqBcqGGHbqycqGG4baEaeqaaOGaeyyXIC9aaSaaaeaacqWFXoqydaWgaaWcbaGaem4qameabeaaaOqaaiab=f7aHnaaBaaaleaacqWGdbWqaeqaaOGaey4kaSIaem4saS0aa0baaSqaaiabdseaebqaaiabdMha5baakiabgwSixlab=n7aNbaacqGG7aWoaeaacqWG6bGEdaWgaaWcbaGaeyOhIukabeaakiabg2da9iabdQha6naaBaaaleaacyGGTbqBcqGGHbqycqGG4baEaeqaaOGaeyyXIC9aaSaaaeaacqWFXoqydaWgaaWcbaGaem4qameabeaaaOqaaiab=f7aHnaaBaaaleaacqWGdbWqaeqaaOGaey4kaSIaem4saS0aa0baaSqaaiabdseaebqaaiabdQha6baakiabgwSixlab=n7aNbaacqGG7aWoaaaaaa@7255@

Thus, the steady-state solutions for free calcium and calcium-bound indicator remain the same, no matter whether there is a buffer or not.

In the linear regime, the above equations reduce to the following system, now written in matrix notation for the sake of clarity:

(25)(x′(t)y′(t)z′(t))=[−γ−kfyymax⁡−kfzzmax⁡+kby+kbz+kfyymax⁡−kby0+kfzzmax⁡0−kbz]⋅(x(t)y(t)z(t))+(α(t)00)
 MathType@MTEF@5@5@+=feaafiart1ev1aaatCvAUfKttLearuWrP9MDH5MBPbIqV92AaeXatLxBI9gBaebbnrfifHhDYfgasaacH8akY=wiFfYdH8Gipec8Eeeu0xXdbba9frFj0=OqFfea0dXdd9vqai=hGuQ8kuc9pgc9s8qqaq=dirpe0xb9q8qiLsFr0=vr0=vr0dc8meaabaqaciaacaGaaeqabaqabeGadaaakeaafaqabeqacaaabaWaaeWaaeaacqaIYaGmcqaI1aqnaiaawIcacaGLPaaaaeaadaqadaqaauaabeqadeaaaeaacuWG4baEgaqbaiabcIcaOiabdsha0jabcMcaPaqaaiqbdMha5zaafaGaeiikaGIaemiDaqNaeiykaKcabaGafmOEaONbauaacqGGOaakcqWG0baDcqGGPaqkaaaacaGLOaGaayzkaaGaeyypa0ZaamWaaeaafaqabeWadaaabaGaeyOeI0ccciGae83SdCMaeyOeI0Iaem4AaS2aa0baaSqaaiabdAgaMbqaaiabdMha5baakiabdMha5naaBaaaleaacyGGTbqBcqGGHbqycqGG4baEaeqaaOGaeyOeI0Iaem4AaS2aa0baaSqaaiabdAgaMbqaaiabdQha6baakiabdQha6naaBaaaleaacyGGTbqBcqGGHbqycqGG4baEaeqaaaGcbaGaey4kaSIaem4AaS2aa0baaSqaaiabdkgaIbqaaiabdMha5baaaOqaaiabgUcaRiabdUgaRnaaDaaaleaacqWGIbGyaeaacqWG6bGEaaaakeaacqGHRaWkcqWGRbWAdaqhaaWcbaGaemOzaygabaGaemyEaKhaaOGaemyEaK3aaSbaaSqaaiGbc2gaTjabcggaHjabcIha4bqabaaakeaacqGHsislcqWGRbWAdaqhaaWcbaGaemOyaigabaGaemyEaKhaaaGcbaGaeGimaadabaGaey4kaSIaem4AaS2aa0baaSqaaiabdAgaMbqaaiabdQha6baakiabdQha6naaBaaaleaacyGGTbqBcqGGHbqycqGG4baEaeqaaaGcbaGaeGimaadabaGaeyOeI0Iaem4AaS2aa0baaSqaaiabdkgaIbqaaiabdQha6baaaaaakiaawUfacaGLDbaacqGHflY1daqadaqaauaabeqadeaaaeaacqWG4baEcqGGOaakcqWG0baDcqGGPaqkaeaacqWG5bqEcqGGOaakcqWG0baDcqGGPaqkaeaacqWG6bGEcqGGOaakcqWG0baDcqGGPaqkaaaacaGLOaGaayzkaaGaey4kaSYaaeWaaeaafaqabeWabaaabaGae8xSdeMaeiikaGIaemiDaqNaeiykaKcabaGaeGimaadabaGaeGimaadaaaGaayjkaiaawMcaaaaaaaa@A3CB@

The homogeneous part of this equation has the solutions *k*·*e*^*λt*^, where *λ *is an Eigenvalue of the matrix, and k the respective Eigenvector. The time-constants can, again, be obtained analytically from the characteristic (cubic) equation of the above matrix. The resulting expressions, however, are extremely lengthy and do not give any insight into the solution.

As a further step, we can also study the approximate solution in the nonlinear regime including an internal buffer. We use again the relationship from eq. (17):

(26)y(t)=ymax⁡⋅x(t)KDy+x(t)
 MathType@MTEF@5@5@+=feaafiart1ev1aaatCvAUfKttLearuWrP9MDH5MBPbIqV92AaeXatLxBI9gBaebbnrfifHhDYfgasaacH8akY=wiFfYdH8Gipec8Eeeu0xXdbba9frFj0=OqFfea0dXdd9vqai=hGuQ8kuc9pgc9s8qqaq=dirpe0xb9q8qiLsFr0=vr0=vr0dc8meaabaqaciaacaGaaeqabaqabeGadaaakeaafaqabeqacaaabaWaaeWaaeaacqaIYaGmcqaI2aGnaiaawIcacaGLPaaaaeaacqWG5bqEcqGGOaakcqWG0baDcqGGPaqkcqGH9aqpcqWG5bqEdaWgaaWcbaGagiyBa0MaeiyyaeMaeiiEaGhabeaakiabgwSixpaalaaabaGaemiEaGNaeiikaGIaemiDaqNaeiykaKcabaGaem4saS0aa0baaSqaaiabdseaebqaaiabdMha5baakiabgUcaRiabdIha4jabcIcaOiabdsha0jabcMcaPaaaaaaaaa@4BFF@ and

(27)z(t)=zmax⁡⋅x(t)KDz+x(t)
 MathType@MTEF@5@5@+=feaafiart1ev1aaatCvAUfKttLearuWrP9MDH5MBPbIqV92AaeXatLxBI9gBaebbnrfifHhDYfgasaacH8akY=wiFfYdH8Gipec8Eeeu0xXdbba9frFj0=OqFfea0dXdd9vqai=hGuQ8kuc9pgc9s8qqaq=dirpe0xb9q8qiLsFr0=vr0=vr0dc8meaabaqaciaacaGaaeqabaqabeGadaaakeaafaqabeqacaaabaWaaeWaaeaacqaIYaGmcqaI3aWnaiaawIcacaGLPaaaaeaacqWG6bGEcqGGOaakcqWG0baDcqGGPaqkcqGH9aqpcqWG6bGEdaWgaaWcbaGagiyBa0MaeiyyaeMaeiiEaGhabeaakiabgwSixpaalaaabaGaemiEaGNaeiikaGIaemiDaqNaeiykaKcabaGaem4saS0aa0baaSqaaiabdseaebqaaiabdQha6baakiabgUcaRiabdIha4jabcIcaOiabdsha0jabcMcaPaaaaaaaaa@4C07@

From eq. (27), obtain the derivative z'(t):

(28)z′(t)=zmax⁡⋅x′(t)⋅KDz(KDz+x(t))2
 MathType@MTEF@5@5@+=feaafiart1ev1aaatCvAUfKttLearuWrP9MDH5MBPbIqV92AaeXatLxBI9gBaebbnrfifHhDYfgasaacH8akY=wiFfYdH8Gipec8Eeeu0xXdbba9frFj0=OqFfea0dXdd9vqai=hGuQ8kuc9pgc9s8qqaq=dirpe0xb9q8qiLsFr0=vr0=vr0dc8meaabaqaciaacaGaaeqabaqabeGadaaakeaafaqabeqacaaabaWaaeWaaeaacqaIYaGmcqaI4aaoaiaawIcacaGLPaaaaeaacuWG6bGEgaqbaiabcIcaOiabdsha0jabcMcaPiabg2da9iabdQha6naaBaaaleaacyGGTbqBcqGGHbqycqGG4baEaeqaaOGaeyyXIC9aaSaaaeaacuWG4baEgaqbaiabcIcaOiabdsha0jabcMcaPiabgwSixlabdUealnaaDaaaleaacqWGebaraeaacqWG6bGEaaaakeaadaqadaqaaiabdUealnaaDaaaleaacqWGebaraeaacqWG6bGEaaGccqGHRaWkcqWG4baEcqGGOaakcqWG0baDcqGGPaqkaiaawIcacaGLPaaadaahaaWcbeqaaiabikdaYaaaaaaaaaaa@54F7@

We rearrange eq. (26) to obtain

(29)x(t)=KDy⋅y(t)ymax⁡−y(t)
 MathType@MTEF@5@5@+=feaafiart1ev1aaatCvAUfKttLearuWrP9MDH5MBPbIqV92AaeXatLxBI9gBaebbnrfifHhDYfgasaacH8akY=wiFfYdH8Gipec8Eeeu0xXdbba9frFj0=OqFfea0dXdd9vqai=hGuQ8kuc9pgc9s8qqaq=dirpe0xb9q8qiLsFr0=vr0=vr0dc8meaabaqaciaacaGaaeqabaqabeGadaaakeaafaqabeqacaaabaWaaeWaaeaacqaIYaGmcqaI5aqoaiaawIcacaGLPaaaaeaacqWG4baEcqGGOaakcqWG0baDcqGGPaqkcqGH9aqpcqWGlbWsdaqhaaWcbaGaemiraqeabaGaemyEaKhaaOGaeyyXIC9aaSaaaeaacqWG5bqEcqGGOaakcqWG0baDcqGGPaqkaeaacqWG5bqEdaWgaaWcbaGagiyBa0MaeiyyaeMaeiiEaGhabeaakiabgkHiTiabdMha5jabcIcaOiabdsha0jabcMcaPaaaaaaaaa@4C12@, and from that, calculate x'(t):

(30)x′(t)=KDy⋅ymax⁡y′(t)(ymax⁡−y(t))2.
 MathType@MTEF@5@5@+=feaafiart1ev1aaatCvAUfKttLearuWrP9MDH5MBPbIqV92AaeXatLxBI9gBaebbnrfifHhDYfgasaacH8akY=wiFfYdH8Gipec8Eeeu0xXdbba9frFj0=OqFfea0dXdd9vqai=hGuQ8kuc9pgc9s8qqaq=dirpe0xb9q8qiLsFr0=vr0=vr0dc8meaabaqaciaacaGaaeqabaqabeGadaaakeaafaqabeqacaaabaWaaeWaaeaacqaIZaWmcqaIWaamaiaawIcacaGLPaaaaeaacuWG4baEgaqbaiabcIcaOiabdsha0jabcMcaPiabg2da9iabdUealnaaDaaaleaacqWGebaraeaacqWG5bqEaaGccqGHflY1cqWG5bqEdaWgaaWcbaGagiyBa0MaeiyyaeMaeiiEaGhabeaakmaalaaabaGafmyEaKNbauaacqGGOaakcqWG0baDcqGGPaqkaeaadaqadaqaaiabdMha5naaBaaaleaacyGGTbqBcqGGHbqycqGG4baEaeqaaOGaeyOeI0IaemyEaKNaeiikaGIaemiDaqNaeiykaKcacaGLOaGaayzkaaWaaWbaaSqabeaacqaIYaGmaaaaaOGaeiOla4caaaaa@5587@

Using eqs. (29) and (30), we now substitute x(t) and x'(t) in eq. (28) and obtain:

(31)z′(t)=zmax⁡⋅KDy⋅ymax⁡y′(t)(ymax⁡−y(t))2⋅KDz⋅1(KDz+KDy⋅y(t)ymax⁡−y(t))2
 MathType@MTEF@5@5@+=feaafiart1ev1aaatCvAUfKttLearuWrP9MDH5MBPbIqV92AaeXatLxBI9gBaebbnrfifHhDYfgasaacH8akY=wiFfYdH8Gipec8Eeeu0xXdbba9frFj0=OqFfea0dXdd9vqai=hGuQ8kuc9pgc9s8qqaq=dirpe0xb9q8qiLsFr0=vr0=vr0dc8meaabaqaciaacaGaaeqabaqabeGadaaakeaafaqabeqacaaabaWaaeWaaeaacqaIZaWmcqaIXaqmaiaawIcacaGLPaaaaeaacuWG6bGEgaqbaiabcIcaOiabdsha0jabcMcaPiabg2da9iabdQha6naaBaaaleaacyGGTbqBcqGGHbqycqGG4baEaeqaaOGaeyyXICTaem4saS0aa0baaSqaaiabdseaebqaaiabdMha5baakiabgwSixlabdMha5naaBaaaleaacyGGTbqBcqGGHbqycqGG4baEaeqaaOWaaSaaaeaacuWG5bqEgaqbaiabcIcaOiabdsha0jabcMcaPaqaamaabmaabaGaemyEaK3aaSbaaSqaaiGbc2gaTjabcggaHjabcIha4bqabaGccqGHsislcqWG5bqEcqGGOaakcqWG0baDcqGGPaqkaiaawIcacaGLPaaadaahaaWcbeqaaiabikdaYaaaaaGccqGHflY1cqWGlbWsdaqhaaWcbaGaemiraqeabaGaemOEaOhaaOGaeyyXIC9aaSaaaeaacqaIXaqmaeaadaqadaqaaiabdUealnaaDaaaleaacqWGebaraeaacqWG6bGEaaGccqGHRaWkcqWGlbWsdaqhaaWcbaGaemiraqeabaGaemyEaKhaaOGaeyyXIC9aaSaaaeaacqWG5bqEcqGGOaakcqWG0baDcqGGPaqkaeaacqWG5bqEdaWgaaWcbaGagiyBa0MaeiyyaeMaeiiEaGhabeaakiabgkHiTiabdMha5jabcIcaOiabdsha0jabcMcaPaaaaiaawIcacaGLPaaadaahaaWcbeqaaiabikdaYaaaaaaaaaaa@83EE@

Now, we use eqs. (29), (30) and (31) and substitute in eq. (21). Rearranging for *α*(t) gives:

(32)α(t)=γ⋅KDy⋅y(t)ymax⁡−y(t)+y′(t)⋅[1+KDy⋅ymax⁡(ymax⁡−y(t))2+KDy⋅ymax⁡⋅KDz⋅zmax⁡(y(t)⋅(KDz−KDy)+KDz⋅ymax⁡)2]
 MathType@MTEF@5@5@+=feaafiart1ev1aaatCvAUfKttLearuWrP9MDH5MBPbIqV92AaeXatLxBI9gBaebbnrfifHhDYfgasaacH8akY=wiFfYdH8Gipec8Eeeu0xXdbba9frFj0=OqFfea0dXdd9vqai=hGuQ8kuc9pgc9s8qqaq=dirpe0xb9q8qiLsFr0=vr0=vr0dc8meaabaqaciaacaGaaeqabaqabeGadaaakeaafaqabeqacaaabaWaaeWaaeaacqaIZaWmcqaIYaGmaiaawIcacaGLPaaaaeaaiiGacqWFXoqycqGGOaakcqWG0baDcqGGPaqkcqGH9aqpdaWcaaqaaiab=n7aNjabgwSixlabdUealnaaDaaaleaacqWGebaraeaacqWG5bqEaaGccqGHflY1cqWG5bqEcqGGOaakcqWG0baDcqGGPaqkaeaacqWG5bqEdaWgaaWcbaGagiyBa0MaeiyyaeMaeiiEaGhabeaakiabgkHiTiabdMha5jabcIcaOiabdsha0jabcMcaPaaacqGHRaWkcuWG5bqEgaqbaiabcIcaOiabdsha0jabcMcaPiabgwSixpaadmaabaGaeGymaeJaey4kaSYaaSaaaeaacqWGlbWsdaqhaaWcbaGaemiraqeabaGaemyEaKhaaOGaeyyXICTaemyEaK3aaSbaaSqaaiGbc2gaTjabcggaHjabcIha4bqabaaakeaadaqadaqaaiabdMha5naaBaaaleaacyGGTbqBcqGGHbqycqGG4baEaeqaaOGaeyOeI0IaemyEaKNaeiikaGIaemiDaqNaeiykaKcacaGLOaGaayzkaaWaaWbaaSqabeaacqaIYaGmaaaaaOGaey4kaSYaaSaaaeaacqWGlbWsdaqhaaWcbaGaemiraqeabaGaemyEaKhaaOGaeyyXICTaemyEaK3aaSbaaSqaaiGbc2gaTjabcggaHjabcIha4bqabaGccqGHflY1cqWGlbWsdaqhaaWcbaGaemiraqeabaGaemOEaOhaaOGaeyyXICTaemOEaO3aaSbaaSqaaiGbc2gaTjabcggaHjabcIha4bqabaaakeaadaqadaqaaiabdMha5jabcIcaOiabdsha0jabcMcaPiabgwSixpaabmaabaGaem4saS0aa0baaSqaaiabdseaebqaaiabdQha6baakiabgkHiTiabdUealnaaDaaaleaacqWGebaraeaacqWG5bqEaaaakiaawIcacaGLPaaacqGHRaWkcqWGlbWsdaqhaaWcbaGaemiraqeabaGaemOEaOhaaOGaeyyXICTaemyEaK3aaSbaaSqaaiGbc2gaTjabcggaHjabcIha4bqabaaakiaawIcacaGLPaaadaahaaWcbeqaaiabikdaYaaaaaaakiaawUfacaGLDbaaaaaaaa@B1E3@

Thus, the calcium influx can be determined in a manner similar to the situation without such a buffer (note how eq. (32) reduces to eq. (19) when z_max _becomes zero). In order to do so, one also has to know the total amount of calcium-bound and free internal buffer plus its binding constant.

Removing the indicator y(t) from eq. (21) and inserting eq. (28), the unperturbed calcium concentration x*(t) is the solution of the following nonlinear differential equation:

(33)x∗′(t)=(α(t)−γ⋅x∗(t))⋅[1+zmax⁡⋅KDz(KDz+x∗(t))2]−1
 MathType@MTEF@5@5@+=feaafiart1ev1aaatCvAUfKttLearuWrP9MDH5MBPbIqV92AaeXatLxBI9gBaebbnrfifHhDYfgasaacH8akY=wiFfYdH8Gipec8Eeeu0xXdbba9frFj0=OqFfea0dXdd9vqai=hGuQ8kuc9pgc9s8qqaq=dirpe0xb9q8qiLsFr0=vr0=vr0dc8meaabaqaciaacaGaaeqabaqabeGadaaakeaafaqabeqacaaabaWaaeWaaeaacqaIZaWmcqaIZaWmaiaawIcacaGLPaaaaeaacqWG4baEdaahaaWcbeqaaiabgEHiQOGamai0gkdiIcaacqGGOaakcqWG0baDcqGGPaqkcqGH9aqpdaqadaqaaGGaciab=f7aHjabcIcaOiabdsha0jabcMcaPiabgkHiTiab=n7aNjabgwSixlabdIha4naaCaaaleqabaGaey4fIOcaaOGaeiikaGIaemiDaqNaeiykaKcacaGLOaGaayzkaaGaeyyXIC9aamWaaeaacqaIXaqmcqGHRaWkdaWcaaqaaiabdQha6naaBaaaleaacyGGTbqBcqGGHbqycqGG4baEaeqaaOGaeyyXICTaem4saS0aa0baaSqaaiabdseaebqaaiabdQha6baaaOqaamaabmaabaGaem4saS0aa0baaSqaaiabdseaebqaaiabdQha6baakiabgUcaRiabdIha4naaCaaaleqabaGaey4fIOcaaOGaeiikaGIaemiDaqNaeiykaKcacaGLOaGaayzkaaWaaWbaaSqabeaacqaIYaGmaaaaaaGccaGLBbGaayzxaaWaaWbaaSqabeaacqGHsislcqaIXaqmaaaaaaaa@6C20@

This equation can be solved by numerical integration. Note again that eq. (33) reduces to eq. (16) when z_max _becomes zero.

## Discussion

In the work presented above we have derived, from first principles, the dependence of the time-course of the indicator signal on the calcium influx and the relevant properties of the indicator and the cell under investigation. In order to do so, we assumed that the system approximately follows its steady-state at every point in time (eq. (17)). Under these conditions, we were able to calculate the calcium influx from the indicator time-course, no matter whether the free calcium concentration is in the linear or nonlinear range with respect to the binding constant of the indicator. Ignoring a cell-internal buffer system, this solution is represented by our eq. (19), from which the time-course of the unperturbed calcium concentration can be derived by a simple convolution with a 1^st ^order low-pass filter, the time-constant of which is given by the inverse of the pump rate, i.e. 1/*γ*. Importantly, by using perturbation analysis, we were also able to indicate the parameter regime within which this solution is valid.

We also included an additional cell internal buffer in our model. Using the same approximation as above, i.e. eq. (17), we could calculate the calcium influx from the indicator time-course (eq. (32)) and the time-course of the unperturbed calcium concentration under these conditions (eq. (33)). In contrast to the situation without internal buffer, the unperturbed calcium concentration does not follow the calcium influx as fed through a linear, 1^st ^order low-pass filter but, instead, is altered by the dynamic interaction to and from the cell-internal buffer. In this case, however, we could not indicate the parameter range within which our solution is valid.

It is straightforward to see how the above approach can be extended to include several buffer systems. Nevertheless, our current analysis ignores some of the complexity that real nerve cells exhibit, such as feed-back of the intracellular calcium level on to the membrane currents via calcium-dependent Ca- and K-conductances. While these can be included in numerical simulations of calcium dynamics, analytical treatment of the resulting equations are beyond the scope of the present paper and have to await future investigation.

### Feasibility of the approach

To apply the approach outlined above to an experimental situation, one has to realize, first of all, that the indicator bound calcium (y(t) in our terminology) is not a parameter immediately being measured. Instead, what is immediately measured is a fluorescence signal. This is, of course, related to the indicator bound calcium, and the quantification of this relationship is given in Appendix II. Nevertheless, the application of our approach to an experimental situation, in particular in the nonlinear regime, has some shortcomings. First of all, application of eqs. (15) or (19) requires knowledge of parameters such as extrusion rate, initial indicator concentration etc. If these are not known, the calcium influx can-not be calculated. But even if all these parameters are known, the application of eqs. (15) or (19) is problematic since the indicator signal will be subject to noise. In this event, taking the first or second order derivatives of a measured signal will boost the noise, and-, dividing by small values of (*y*_max _- *y*(*t*))^2 ^(when the bound indicator is saturating, i.e. approaching the initial free indicator concentration) will further lead to unstable solutions. Therefore, alternative approaches should be considered.

### Alternative approach I: linear regime

One such alternative approach is applicable when the relationship between the membrane voltage and the calcium influx and the indicator signal is linear through all stages. While the first relationship, i.e. the one between membrane voltage and calcium influx, is in general not linear, one can either work with small membrane deviations around a potential where calcium channels are already activated, or use the number of action potentials of the actual membrane potential as the signal V(t). The method outlined below requires measuring the voltage signal *V*(*t*) and indicator signal y(t) simultaneously. Then we can determine the relationship between the voltage and the bound indicator time course, and from the latter determine *V*(*t*). If we know *V*(*t*), we can use an equation for calcium dynamics to predict the calcium influx *α*(*t*).

In the linear regime we can do this by assuming that *y*(*t*) is given by a first order kernel *g*(*t*) in terms of *V*(*t*)

(34)     *y*(*t*) = ∫*dt' g*(*t *- *t'*)*V *(*t'*).

From several such example recordings, the optimal reverse filter g_rev_(t) can be calculated in the Fourier domain using the Wiener-Kolmogorov formalism if the calcium concentrations are small compared to the K_D _value of the indicator, i.e. when the system is in the linear regime. Under these conditions, the bound indicator concentration can be calculated from the calcium influx as a convolution with the following so-called 'forward' filter g_forw_(t) (see eq. (13)):

(35)gforw(t)=ymax⁡2⋅γ⋅KD⋅[(1+AA2−4kbγ)⋅exp⁡(−t/τ1)+(1−AA2−4kbγ)⋅exp⁡(−t/τ2)]
 MathType@MTEF@5@5@+=feaafiart1ev1aaatCvAUfKttLearuWrP9MDH5MBPbIqV92AaeXatLxBI9gBaebbnrfifHhDYfgasaacH8akY=wiFfYdH8Gipec8Eeeu0xXdbba9frFj0=OqFfea0dXdd9vqai=hGuQ8kuc9pgc9s8qqaq=dirpe0xb9q8qiLsFr0=vr0=vr0dc8meaabaqaciaacaGaaeqabaqabeGadaaakeaafaqabeqacaaabaWaaeWaaeaacqaIZaWmcqaI1aqnaiaawIcacaGLPaaaaeaacqWGNbWzdaWgaaWcbaGaemOzayMaem4Ba8MaemOCaiNaem4DaChabeaakiabcIcaOiabdsha0jabcMcaPiabg2da9maalaaabaGaemyEaK3aaSbaaSqaaiGbc2gaTjabcggaHjabcIha4bqabaaakeaacqaIYaGmcqGHflY1iiGacqWFZoWzcqGHflY1cqWGlbWsdaWgaaWcbaGaemiraqeabeaaaaGccqGHflY1daWadaqaamaabmaabaGaeGymaeJaey4kaSYaaSaaaeaacqWGbbqqaeaadaGcaaqaaiabdgeabnaaCaaaleqabaGaeGOmaidaaOGaeyOeI0IaeGinaqJaem4AaS2aaSbaaSqaaiabdkgaIbqabaGccqWFZoWzaSqabaaaaaGccaGLOaGaayzkaaGaeyyXICTagiyzauMaeiiEaGNaeiiCaa3aaeWaaeaacqGHsislcqWG0baDcqGGVaWlcqWFepaDdaWgaaWcbaGaeGymaedabeaaaOGaayjkaiaawMcaaiabgUcaRmaabmaabaGaeGymaeJaeyOeI0YaaSaaaeaacqWGbbqqaeaadaGcaaqaaiabdgeabnaaCaaaleqabaGaeGOmaidaaOGaeyOeI0IaeGinaqJaem4AaS2aaSbaaSqaaiabdkgaIbqabaGccqWFZoWzaSqabaaaaaGccaGLOaGaayzkaaGaeyyXICTagiyzauMaeiiEaGNaeiiCaa3aaeWaaeaacqGHsislcqWG0baDcqGGVaWlcqWFepaDdaWgaaWcbaGaeGOmaidabeaaaOGaayjkaiaawMcaaaGaay5waiaaw2faaaaaaaa@86FE@

Given that there is a linear relationship between membrane voltage and calcium influx, the problem of recovering the membrane voltage from indicator measurements is to find the optimal reverse filter, which can then be applied to all those situations where only the optical signal from the calcium-bound indicator y(t) has been measured. As can be shown, the optimal reverse filter g_rev_(t) is not the inverse of the forward filter that turns V(t) into y(t) (as done by Yaksi and Friedrich, [9]), but rather the average cross-correlation between V(t) and y(t), divided by the power spectrum of y(t) [10,11]. Denoting the inverse Fourier Transform by F^-1^, y*(f) the complex conjugate of y(f) and the average across n trials by ⟨...⟩, the optimal reverse filter g_rev_(t) becomes:

(36)grev(t)=F−1{g(f)}=F−1{〈Vi(f)⋅yi∗(f)〉〈yi(f)⋅yi(f)〉}
 MathType@MTEF@5@5@+=feaafiart1ev1aaatCvAUfKttLearuWrP9MDH5MBPbIqV92AaeXatLxBI9gBaebbnrfifHhDYfgasaacH8akY=wiFfYdH8Gipec8Eeeu0xXdbba9frFj0=OqFfea0dXdd9vqai=hGuQ8kuc9pgc9s8qqaq=dirpe0xb9q8qiLsFr0=vr0=vr0dc8meaabaqaciaacaGaaeqabaqabeGadaaakeaafaqabeqacaaabaWaaeWaaeaacqaIZaWmcqaI2aGnaiaawIcacaGLPaaaaeaacqWGNbWzdaWgaaWcbaGaemOCaiNaemyzauMaemODayhabeaakiabcIcaOiabdsha0jabcMcaPiabg2da9iabdAeagnaaCaaaleqabaGaeyOeI0IaeGymaedaaOGaei4EaSNaem4zaCMaeiikaGIaemOzayMaeiykaKIaeiyFa0Naeyypa0JaemOray0aaWbaaSqabeaacqGHsislcqaIXaqmaaGcdaGadeqaamaalaaabaWaaaWabeaacqWGwbGvdaWgaaWcbaGaemyAaKgabeaakiabcIcaOiabdAgaMjabcMcaPiabgwSixlabdMha5naaDaaaleaacqWGPbqAaeaacqGHxiIkaaGccqGGOaakcqWGMbGzcqGGPaqkaiaawMYicaGLQmcaaeaadaaadeqaaiabdMha5naaBaaaleaacqWGPbqAaeqaaOGaeiikaGIaemOzayMaeiykaKIaeyyXICTaemyEaK3aaSbaaSqaaiabdMgaPbqabaGccqGGOaakcqWGMbGzcqGGPaqkaiaawMYicaGLQmcaaaaacaGL7bGaayzFaaaaaaaa@6C3F@

**Figure 3:**
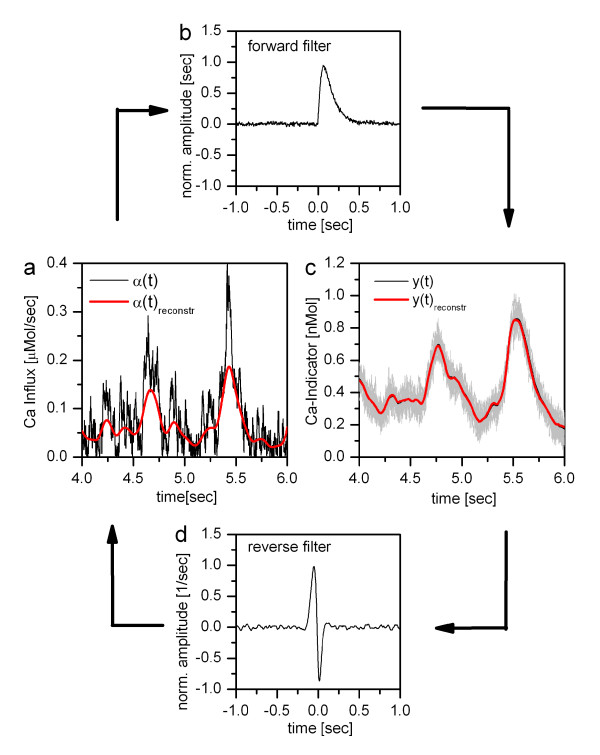
Reverse reconstruction of the calcium influx from the indicator signal. **a**: Calcium influx *α*(t) (in black) together with the reconstructed influx (in red). **b**: Optimal forward filter g_forw_(t). **c**: Average time course of calcium-bound indicator y(t) (in black), reconstructed signal (in red), and 12 individual indicator signals (in grey). **d**: Optimal reverse filter g_rev_(t).

Convolving each new optical signal y(t) with g_rev_(t) then results in the optimal estimate of the voltage signal, leading to a calcium influx and consequently to the optical signal of bound indicator. Clearly, the advantage of this method is that no parameters need to be known; the disadvantage is that enough dual measurements of membrane voltage and indicator need to be at hand to calculate the optimal reverse filter g_rev_(t). As another caveat, this method only works as long as calcium concentrations are in the linear regime with respect to the K_D _of the indicator and to membrane voltage. An example of a reverse reconstruction in the linear regime is shown in Fig. 3. Here, the signal was created by Gaussian noise with an auto-correlation time-constant of 100 ms that was subsequently rectified. From this influx, the calcium bound indicator concentration y(t) was numerically determined using eqs. (1) and (2) and the following parameters: pump rate *γ *= 20 Hz, k_f _= 0.01 1/(nMol sec), k_b _= 10 Hz, resulting in a K_D _value of 1000 nMol, and an initial indicator concentration y_max _= 100 nMol. This led to the average time course of calcium-bound indicator y(t) shown as a black line in Fig. 3c. Through 100 trials, a Gaussian noise signal was added with an auto-correlation time-constant of 10 ms, which had an average amplitude of 5% of y(t). Twelve such trials are shown as grey lines superimposed on Fig. 3c. From these trials, optimal forward g_forw_(t) and reverse filter g_rev_(t) were calculated according to gforw(t)=F−1{〈yi(f)⋅αi∗(f)〉/〈αi(f)⋅αi∗(f)〉}
 MathType@MTEF@5@5@+=feaafiart1ev1aaatCvAUfKttLearuWrP9MDH5MBPbIqV92AaeXatLxBI9gBaebbnrfifHhDYfgasaacH8akY=wiFfYdH8Gipec8Eeeu0xXdbba9frFj0=OqFfea0dXdd9vqai=hGuQ8kuc9pgc9s8qqaq=dirpe0xb9q8qiLsFr0=vr0=vr0dc8meaabaqaciaacaGaaeqabaqabeGadaaakeaacqWGNbWzdaWgaaWcbaGaemOzayMaem4Ba8MaemOCaiNaem4DaChabeaakiabcIcaOiabdsha0jabcMcaPiabg2da9iabdAeagnaaCaaaleqabaGaeyOeI0IaeGymaedaaOWaaiWabeaadaaadeqaaiabdMha5naaBaaaleaacqWGPbqAaeqaaOGaeiikaGIaemOzayMaeiykaKIaeyyXICncciGae8xSde2aa0baaSqaaiabdMgaPbqaaiabgEHiQaaakiabcIcaOiabdAgaMjabcMcaPaGaayzkJiaawQYiaiabc+caVmaaamqabaGae8xSde2aaSbaaSqaaiabdMgaPbqabaGccqGGOaakcqWGMbGzcqGGPaqkcqGHflY1cqWFXoqydaqhaaWcbaGaemyAaKgabaGaey4fIOcaaOGaeiikaGIaemOzayMaeiykaKcacaGLPmIaayPkJaaacaGL7bGaayzFaaaaaa@610E@ and grev(t)=F−1{〈αi(f)⋅yi∗(f)〉/〈yi(f)⋅yi∗(f)〉}
 MathType@MTEF@5@5@+=feaafiart1ev1aaatCvAUfKttLearuWrP9MDH5MBPbIqV92AaeXatLxBI9gBaebbnrfifHhDYfgasaacH8akY=wiFfYdH8Gipec8Eeeu0xXdbba9frFj0=OqFfea0dXdd9vqai=hGuQ8kuc9pgc9s8qqaq=dirpe0xb9q8qiLsFr0=vr0=vr0dc8meaabaqaciaacaGaaeqabaqabeGadaaakeaacqWGNbWzdaWgaaWcbaGaemOCaiNaemyzauMaemODayhabeaakiabcIcaOiabdsha0jabcMcaPiabg2da9iabdAeagnaaCaaaleqabaGaeyOeI0IaeGymaedaaOWaaiWabeaadaaadeqaaGGaciab=f7aHnaaBaaaleaacqWGPbqAaeqaaOGaeiikaGIaemOzayMaeiykaKIaeyyXICTaemyEaK3aa0baaSqaaiabdMgaPbqaaiabgEHiQaaakiabcIcaOiabdAgaMjabcMcaPaGaayzkJiaawQYiaiabc+caVmaaamqabaGaemyEaK3aaSbaaSqaaiabdMgaPbqabaGccqGGOaakcqWGMbGzcqGGPaqkcqGHflY1cqWG5bqEdaqhaaWcbaGaemyAaKgabaGaey4fIOcaaOGaeiikaGIaemOzayMaeiykaKcacaGLPmIaayPkJaaacaGL7bGaayzFaaaaaa@5F65@. These filters are shown in b and d, respectively. Applying the forward filter to *α*(t), the signal shown in red in Fig. 3c was obtained. Applying the reverse filter to y(t), the signal shown in red in Fig. 3a was obtained. Note that while the forward filter leads to an output that is almost indistinguishable from y(t), the reverse filter can only reconstruct the low-frequency components of *α*(t), since high frequency components are covered by noise in the individual response trials.

### Alternative approach II: nonlinear regime

If either of the two relationships, i.e. the one between membrane voltage V(t) and the calcium influx *α*(t) or the one between *α*(t) and bound indicator y(t) (due to a high calcium level with respect to the K_D _value of the indicator) is nonlinear, the above method will lead to erroneous results. In such a nonlinear regime we must use a different approach. Here, too, we must measure the indicator signal *y*(*t*) and the membrane voltage *V*(*t*) simultaneously. In the reconstructed state space [12] of the voltage measurement we can fully describe the state of the system (neuron plus indicator) using the voltage and its time lags, or we can use the indicator signal and its time lags. If we do the latter, we create data vectors

(37)     *U*(*t*) = [*y*(*t*), *y*(*t *- *τ*), *y*(*t *- 2*τ*), ..., *y*(*t *- (*D *- 1)*τ*)],

where the number of lags *D *and the time lag *τ *are respectively determined by the method of false nearest neighbors and by average mutual information. For every *U*(*t*) there is an associated indicator signal *V*(*t*), and since we have already totally characterized the state of the neuron by *U*(*t*) there must be a nonlinear relationship *V*(*t*) = *f*(*U*(*t*)). We can discover this nonlinear relation from the simultaneous measurements of *y*(*t*) and *V*(*t*), then, just as in the linear case, map new measurements of *y*(*t*) to allow us to predict *V*(*t*).

The method requires determining *f*(*U*(*t*)). To accomplish this, we represent *f*(*U*) in terms of some basis functions chosen by the user: *φ*_*m*_(*U*), and write

(38)f(U)=∑m=1Mcmϕm(U).
 MathType@MTEF@5@5@+=feaafiart1ev1aaatCvAUfKttLearuWrP9MDH5MBPbIqV92AaeXatLxBI9gBaebbnrfifHhDYfgasaacH8akY=wiFfYdH8Gipec8Eeeu0xXdbba9frFj0=OqFfea0dXdd9vqai=hGuQ8kuc9pgc9s8qqaq=dirpe0xb9q8qiLsFr0=vr0=vr0dc8meaabaqaciaacaGaaeqabaqabeGadaaakeaafaqabeqacaaabaWaaeWaaeaacqaIZaWmcqaI4aaoaiaawIcacaGLPaaaaeaacqWGMbGzcqGGOaakcqWGvbqvcqGGPaqkcqGH9aqpdaaeWbqaaiabdogaJnaaBaaaleaacqWGTbqBaeqaaGGacOGae8x1dO2aaSbaaSqaaiabd2gaTbqabaGccqGGOaakcqWGvbqvcqGGPaqkaSqaaiabd2gaTjabg2da9iabigdaXaqaaiabd2eanbqdcqGHris5aOGaeiOla4caaaaa@4659@

In the state space of the *U's*, each state vector has many neighbors *U*^(*l*)^(*t*); *l *= 0,1...,*N*_*B*_; *U*^(0)^(*t*) = *U*(*t*). Each of these neighbors maps into a voltage *V*^(*l*)^(*t*) = *f*(*U*^(*l*)^(*t*)). At any given time, corresponding to a location in *U *space, we can determine the coefficients *c*_*m *_by minimizing the least squares form

(39)∑l=0NB(V(l)(t)−∑m=1Mcm(t)ϕm(U(l)(t)))2.
 MathType@MTEF@5@5@+=feaafiart1ev1aaatCvAUfKttLearuWrP9MDH5MBPbIqV92AaeXatLxBI9gBaebbnrfifHhDYfgasaacH8akY=wiFfYdH8Gipec8Eeeu0xXdbba9frFj0=OqFfea0dXdd9vqai=hGuQ8kuc9pgc9s8qqaq=dirpe0xb9q8qiLsFr0=vr0=vr0dc8meaabaqaciaacaGaaeqabaqabeGadaaakeaafaqabeqacaaabaWaaeWaaeaacqaIZaWmcqaI5aqoaiaawIcacaGLPaaaaeaadaaeWbqaamaabmaabaGaemOvay1aaWbaaSqabeaacqGGOaakcqWGSbaBcqGGPaqkaaGccqGGOaakcqWG0baDcqGGPaqkcqGHsisldaaeWbqaaiabdogaJnaaBaaaleaacqWGTbqBaeqaaOGaeiikaGIaemiDaqNaeiykaKccciGae8x1dO2aaSbaaSqaaiabd2gaTbqabaGccqGGOaakcqWGvbqvdaahaaWcbeqaaiabcIcaOiabdYgaSjabcMcaPaaakiabcIcaOiabdsha0jabcMcaPiabcMcaPaWcbaGaemyBa0Maeyypa0JaeGymaedabaGaemyta0eaniabggHiLdaakiaawIcacaGLPaaadaahaaWcbeqaaiabikdaYaaakiabc6caUaWcbaGaemiBaWMaeyypa0JaeGimaadabaGaemOta40aaSbaaWqaaiabdkeacbqabaaaniabggHiLdaaaaaa@5DE2@

This establishes the map *V*(*t*) = *f*(*U*(*t*)) locally in *U *space. Now we make a new measurement of *y*_*new*_(*t*). Use this to create a new D-dimensional data vector *U*_*new*_(*t*) = [*y*_*new*_(*t*), *y*_*new*_(*t *- *τ*), *y*_*new*_(*t *- 2*τ*), ..., *y*_*new*_(*t *- (*D *- 1)*τ*)]. Search among all the data vectors in the initial training set and find the one is closest to *U*_*new*_(*t*); suppose it is *U*(*t'*). Then using the local map attached to *U*(*t'*) we predict

(40)Vnew(t)=∑m=1Mcm(t′)ϕm(Unew(t)).
 MathType@MTEF@5@5@+=feaafiart1ev1aaatCvAUfKttLearuWrP9MDH5MBPbIqV92AaeXatLxBI9gBaebbnrfifHhDYfgasaacH8akY=wiFfYdH8Gipec8Eeeu0xXdbba9frFj0=OqFfea0dXdd9vqai=hGuQ8kuc9pgc9s8qqaq=dirpe0xb9q8qiLsFr0=vr0=vr0dc8meaabaqaciaacaGaaeqabaqabeGadaaakeaafaqabeqacaaabaWaaeWaaeaacqaI0aancqaIWaamaiaawIcacaGLPaaaaeaacqWGwbGvdaWgaaWcbaGaemOBa4MaemyzauMaem4DaChabeaakiabcIcaOiabdsha0jabcMcaPiabg2da9maaqahabaGaem4yam2aaSbaaSqaaiabd2gaTbqabaGccqGGOaakcuWG0baDgaqbaiabcMcaPGGaciab=v9aQnaaBaaaleaacqWGTbqBaeqaaOGaeiikaGIaemyvau1aaSbaaSqaaiabd6gaUjabdwgaLjabdEha3bqabaGccqGGOaakcqWG0baDcqGGPaqkcqGGPaqkcqGGUaGlaSqaaiabd2gaTjabg2da9iabigdaXaqaaiabd2eanbqdcqGHris5aaaaaaa@557B@

From the time course of new measurements *y*_*new*_(*t*), we are thus able to use the learned map to predict the time course of the new membrane voltage *V*_*new*_(*t*), which was our goal.

### Relationship to previous studies

Previous studies on calcium binding mainly considered steady-state situations or the linear case, i.e. that calcium concentrations are small compared to the dissociation constant K_D _of the indicator [13;14]. In particular, a number of studies investigated the diffusion of Calcium ions in the presence of buffers [15-19]. However, none of these interesting papers focused on the time dependent nonlinear kinetics without diffusion or addressed the temporal stability of our eqs. (1) and (2) for the approximate linear or the approximate nonlinear solutions that we derived above.

Our study can be related to these previous investigations when we combine our approximation about the dynamics of the system without internal buffer (eq. (17)) with the condition of small free calcium concentration. Thus, eq. (17) becomes:

(41)y(t)=ymax⁡⋅x(t)KD+x(t)≈x(t)⋅ymax⁡KD
 MathType@MTEF@5@5@+=feaafiart1ev1aaatCvAUfKttLearuWrP9MDH5MBPbIqV92AaeXatLxBI9gBaebbnrfifHhDYfgasaacH8akY=wiFfYdH8Gipec8Eeeu0xXdbba9frFj0=OqFfea0dXdd9vqai=hGuQ8kuc9pgc9s8qqaq=dirpe0xb9q8qiLsFr0=vr0=vr0dc8meaabaqaciaacaGaaeqabaqabeGadaaakeaafaqabeqacaaabaWaaeWaaeaacqaI0aancqaIXaqmaiaawIcacaGLPaaaaeaacqWG5bqEcqGGOaakcqWG0baDcqGGPaqkcqGH9aqpcqWG5bqEdaWgaaWcbaGagiyBa0MaeiyyaeMaeiiEaGhabeaakiabgwSixpaalaaabaGaemiEaGNaeiikaGIaemiDaqNaeiykaKcabaGaem4saS0aaSbaaSqaaiabdseaebqabaGccqGHRaWkcqWG4baEcqGGOaakcqWG0baDcqGGPaqkaaGaeyisISRaemiEaGNaeiikaGIaemiDaqNaeiykaKIaeyyXIC9aaSaaaeaacqWG5bqEdaWgaaWcbaGagiyBa0MaeiyyaeMaeiiEaGhabeaaaOqaaiabdUealnaaBaaaleaacqWGebaraeqaaaaaaaaaaa@5B57@, and

(42)y′(t)≈x′(t)⋅ymax⁡KD
 MathType@MTEF@5@5@+=feaafiart1ev1aaatCvAUfKttLearuWrP9MDH5MBPbIqV92AaeXatLxBI9gBaebbnrfifHhDYfgasaacH8akY=wiFfYdH8Gipec8Eeeu0xXdbba9frFj0=OqFfea0dXdd9vqai=hGuQ8kuc9pgc9s8qqaq=dirpe0xb9q8qiLsFr0=vr0=vr0dc8meaabaqaciaacaGaaeqabaqabeGadaaakeaafaqabeqacaaabaWaaeWaaeaacqaI0aancqaIYaGmaiaawIcacaGLPaaaaeaacuWG5bqEgaqbaiabcIcaOiabdsha0jabcMcaPaaacqGHijYUcuWG4baEgaqbaiabcIcaOiabdsha0jabcMcaPiabgwSixpaalaaabaGaemyEaK3aaSbaaSqaaiGbc2gaTjabcggaHjabcIha4bqabaaakeaacqWGlbWsdaWgaaWcbaGaemiraqeabeaaaaaaaa@45BA@

Inserting eq. (41) into eq. (1) leads to:

(43)x′(t)⋅1γ⋅(1+ymax⁡KD)=α(t)γ−x(t)
 MathType@MTEF@5@5@+=feaafiart1ev1aaatCvAUfKttLearuWrP9MDH5MBPbIqV92AaeXatLxBI9gBaebbnrfifHhDYfgasaacH8akY=wiFfYdH8Gipec8Eeeu0xXdbba9frFj0=OqFfea0dXdd9vqai=hGuQ8kuc9pgc9s8qqaq=dirpe0xb9q8qiLsFr0=vr0=vr0dc8meaabaqaciaacaGaaeqabaqabeGadaaakeaafaqabeqacaaabaWaaeWaaeaacqaI0aancqaIZaWmaiaawIcacaGLPaaaaeaacuWG4baEgaqbaiabcIcaOiabdsha0jabcMcaPiabgwSixpaalaaabaGaeGymaedabaacciGae83SdCgaaiabgwSixpaabmaabaGaeGymaeJaey4kaSYaaSaaaeaacqWG5bqEdaWgaaWcbaGagiyBa0MaeiyyaeMaeiiEaGhabeaaaOqaaiabdUealnaaBaaaleaacqWGebaraeqaaaaaaOGaayjkaiaawMcaaiabg2da9maalaaabaGae8xSdeMaeiikaGIaemiDaqNaeiykaKcabaGae83SdCgaaiabgkHiTiabdIha4jabcIcaOiabdsha0jabcMcaPaaaaaa@54BC@

Eq. (43) describes a 1^st ^order low-pass filter with a time-constant equal to

(44)τ=1γ(1+ymax⁡KD)
 MathType@MTEF@5@5@+=feaafiart1ev1aaatCvAUfKttLearuWrP9MDH5MBPbIqV92AaeXatLxBI9gBaebbnrfifHhDYfgasaacH8akY=wiFfYdH8Gipec8Eeeu0xXdbba9frFj0=OqFfea0dXdd9vqai=hGuQ8kuc9pgc9s8qqaq=dirpe0xb9q8qiLsFr0=vr0=vr0dc8meaabaqaciaacaGaaeqabaqabeGadaaakeaafaqabeqacaaabaWaaeWaaeaacqaI0aancqaI0aanaiaawIcacaGLPaaaaeaaiiGacqWFepaDcqGH9aqpdaWcaaqaaiabigdaXaqaaiab=n7aNbaadaqadaqaaiabigdaXiabgUcaRmaalaaabaGaemyEaK3aaSbaaSqaaiGbc2gaTjabcggaHjabcIha4bqabaaakeaacqWGlbWsdaWgaaWcbaGaemiraqeabeaaaaaakiaawIcacaGLPaaaaaaaaa@414A@

With the indicator concentration being small, the time-constant becomes 1/*γ*. Large indicator concentrations, therefore, increase the time-constant from 1/*γ *to the value indicated by eq. (44).

Repeating the above for the situation with an internal buffer, eq. (42) remains unaltered. In a similar way, we derive from eq. (27)

(45)z(t)≈x(t)⋅zmax⁡KDz
 MathType@MTEF@5@5@+=feaafiart1ev1aaatCvAUfKttLearuWrP9MDH5MBPbIqV92AaeXatLxBI9gBaebbnrfifHhDYfgasaacH8akY=wiFfYdH8Gipec8Eeeu0xXdbba9frFj0=OqFfea0dXdd9vqai=hGuQ8kuc9pgc9s8qqaq=dirpe0xb9q8qiLsFr0=vr0=vr0dc8meaabaqaciaacaGaaeqabaqabeGadaaakeaafaqabeqacaaabaWaaeWaaeaacqaI0aancqaI1aqnaiaawIcacaGLPaaaaeaacqWG6bGEcqGGOaakcqWG0baDcqGGPaqkcqGHijYUcqWG4baEcqGGOaakcqWG0baDcqGGPaqkcqGHflY1daWcaaqaaiabdQha6naaBaaaleaacyGGTbqBcqGGHbqycqGG4baEaeqaaaGcbaGaem4saS0aa0baaSqaaiabdseaebqaaiabdQha6baaaaaaaaaa@472A@, and from that

(46)z′(t)≈x′(t)⋅zmax⁡KDz,
 MathType@MTEF@5@5@+=feaafiart1ev1aaatCvAUfKttLearuWrP9MDH5MBPbIqV92AaeXatLxBI9gBaebbnrfifHhDYfgasaacH8akY=wiFfYdH8Gipec8Eeeu0xXdbba9frFj0=OqFfea0dXdd9vqai=hGuQ8kuc9pgc9s8qqaq=dirpe0xb9q8qiLsFr0=vr0=vr0dc8meaabaqaciaacaGaaeqabaqabeGadaaakeaafaqabeqacaaabaWaaeWaaeaacqaI0aancqaI2aGnaiaawIcacaGLPaaaaeaacuWG6bGEgaqbaiabcIcaOiabdsha0jabcMcaPiabgIKi7kqbdIha4zaafaGaeiikaGIaemiDaqNaeiykaKIaeyyXIC9aaSaaaeaacqWG6bGEdaWgaaWcbaGagiyBa0MaeiyyaeMaeiiEaGhabeaaaOqaaiabdUealnaaDaaaleaacqWGebaraeaacqWG6bGEaaaaaOGaeiilaWcaaaaa@482E@

Substituting eqs. (42) and (46) into eq. (21) gives

(47)x′(t)=α(t)−γ⋅x(t)−x′(t)⋅ymax⁡KDy−x′(t)⋅zmax⁡KDz
 MathType@MTEF@5@5@+=feaafiart1ev1aaatCvAUfKttLearuWrP9MDH5MBPbIqV92AaeXatLxBI9gBaebbnrfifHhDYfgasaacH8akY=wiFfYdH8Gipec8Eeeu0xXdbba9frFj0=OqFfea0dXdd9vqai=hGuQ8kuc9pgc9s8qqaq=dirpe0xb9q8qiLsFr0=vr0=vr0dc8meaabaqaciaacaGaaeqabaqabeGadaaakeaafaqabeqacaaabaWaaeWaaeaacqaI0aancqaI3aWnaiaawIcacaGLPaaaaeaacuWG4baEgaqbaiabcIcaOiabdsha0jabcMcaPiabg2da9GGaciab=f7aHjabcIcaOiabdsha0jabcMcaPiabgkHiTiab=n7aNjabgwSixlabdIha4jabcIcaOiabdsha0jabcMcaPiabgkHiTiqbdIha4zaafaGaeiikaGIaemiDaqNaeiykaKIaeyyXIC9aaSaaaeaacqWG5bqEdaWgaaWcbaGagiyBa0MaeiyyaeMaeiiEaGhabeaaaOqaaiabdUealnaaDaaaleaacqWGebaraeaacqWG5bqEaaaaaOGaeyOeI0IafmiEaGNbauaacqGGOaakcqWG0baDcqGGPaqkcqGHflY1daWcaaqaaiabdQha6naaBaaaleaacyGGTbqBcqGGHbqycqGG4baEaeqaaaGcbaGaem4saS0aa0baaSqaaiabdseaebqaaiabdQha6baaaaaaaaaa@676A@

Rearranging leads to

(48)x′(t)⋅1γ(1+ymax⁡KDy+zmax⁡KDz)=α(t)γ−x(t)
 MathType@MTEF@5@5@+=feaafiart1ev1aaatCvAUfKttLearuWrP9MDH5MBPbIqV92AaeXatLxBI9gBaebbnrfifHhDYfgasaacH8akY=wiFfYdH8Gipec8Eeeu0xXdbba9frFj0=OqFfea0dXdd9vqai=hGuQ8kuc9pgc9s8qqaq=dirpe0xb9q8qiLsFr0=vr0=vr0dc8meaabaqaciaacaGaaeqabaqabeGadaaakeaafaqabeqacaaabaWaaeWaaeaacqaI0aancqaI4aaoaiaawIcacaGLPaaaaeaacuWG4baEgaqbaiabcIcaOiabdsha0jabcMcaPiabgwSixpaalaaabaGaeGymaedabaacciGae83SdCgaamaabmaabaGaeGymaeJaey4kaSYaaSaaaeaacqWG5bqEdaWgaaWcbaGagiyBa0MaeiyyaeMaeiiEaGhabeaaaOqaaiabdUealnaaDaaaleaacqWGebaraeaacqWG5bqEaaaaaOGaey4kaSYaaSaaaeaacqWG6bGEdaWgaaWcbaGagiyBa0MaeiyyaeMaeiiEaGhabeaaaOqaaiabdUealnaaDaaaleaacqWGebaraeaacqWG6bGEaaaaaaGccaGLOaGaayzkaaGaeyypa0ZaaSaaaeaacqWFXoqycqGGOaakcqWG0baDcqGGPaqkaeaacqWFZoWzaaGaeyOeI0IaemiEaGNaeiikaGIaemiDaqNaeiykaKcaaaaa@5EA7@

This, again, describes a 1^st ^order low-pass filter with a time-constant equal to:

(49)τ=1γ(1+ymax⁡KDy+zmax⁡KDz).
 MathType@MTEF@5@5@+=feaafiart1ev1aaatCvAUfKttLearuWrP9MDH5MBPbIqV92AaeXatLxBI9gBaebbnrfifHhDYfgasaacH8akY=wiFfYdH8Gipec8Eeeu0xXdbba9frFj0=OqFfea0dXdd9vqai=hGuQ8kuc9pgc9s8qqaq=dirpe0xb9q8qiLsFr0=vr0=vr0dc8meaabaqaciaacaGaaeqabaqabeGadaaakeaafaqabeqacaaabaWaaeWaaeaacqaI0aancqaI5aqoaiaawIcacaGLPaaaaeaaiiGacqWFepaDcqGH9aqpdaWcaaqaaiabigdaXaqaaiab=n7aNbaadaqadaqaaiabigdaXiabgUcaRmaalaaabaGaemyEaK3aaSbaaSqaaiGbc2gaTjabcggaHjabcIha4bqabaaakeaacqWGlbWsdaqhaaWcbaGaemiraqeabaGaemyEaKhaaaaakiabgUcaRmaalaaabaGaemOEaO3aaSbaaSqaaiGbc2gaTjabcggaHjabcIha4bqabaaakeaacqWGlbWsdaqhaaWcbaGaemiraqeabaGaemOEaOhaaaaaaOGaayjkaiaawMcaaiabc6caUaaaaaa@4E63@

Comparing this result to eq. (44), one can see that internal buffering enlarges the time constant by an additive term, equivalent to the one introduced by the indicator. Eq. (49) is identical to eq. (2) in [20].

Neher and Augustine [21] defined the calcium binding capacity as the ratio of the change in bound indicator concentration over the change in free Calcium:

(50)κ=Δy(t)Δx(t)
 MathType@MTEF@5@5@+=feaafiart1ev1aaatCvAUfKttLearuWrP9MDH5MBPbIqV92AaeXatLxBI9gBaebbnrfifHhDYfgasaacH8akY=wiFfYdH8Gipec8Eeeu0xXdbba9frFj0=OqFfea0dXdd9vqai=hGuQ8kuc9pgc9s8qqaq=dirpe0xb9q8qiLsFr0=vr0=vr0dc8meaabaqaciaacaGaaeqabaqabeGadaaakeaafaqabeqacaaabaWaaeWaaeaacqaI1aqncqaIWaamaiaawIcacaGLPaaaaeaaiiGacqWF6oWAcqGH9aqpdaWcaaqaaiabfs5aejabdMha5jabcIcaOiabdsha0jabcMcaPaqaaiabfs5aejabdIha4jabcIcaOiabdsha0jabcMcaPaaaaaaaaa@3EFD@

For the linear case, i.e. when the calcium concentrations are small compared to the dissociation constant K_D_, this quantity is identical to y_max_/K_D_, as can be derived from eq. (42). For the nonlinear case, i.e. when the calcium concentrations are large compared to the dissociation constant K_D_, we first derive from eq. (17)

(51)y′(t)=ymax⁡⋅x′(t)⋅KD(KD+x(t))2
 MathType@MTEF@5@5@+=feaafiart1ev1aaatCvAUfKttLearuWrP9MDH5MBPbIqV92AaeXatLxBI9gBaebbnrfifHhDYfgasaacH8akY=wiFfYdH8Gipec8Eeeu0xXdbba9frFj0=OqFfea0dXdd9vqai=hGuQ8kuc9pgc9s8qqaq=dirpe0xb9q8qiLsFr0=vr0=vr0dc8meaabaqaciaacaGaaeqabaqabeGadaaakeaafaqabeqacaaabaWaaeWaaeaacqaI1aqncqaIXaqmaiaawIcacaGLPaaaaeaacuWG5bqEgaqbaiabcIcaOiabdsha0jabcMcaPiabg2da9iabdMha5naaBaaaleaacyGGTbqBcqGGHbqycqGG4baEaeqaaOGaeyyXIC9aaSaaaeaacuWG4baEgaqbaiabcIcaOiabdsha0jabcMcaPiabgwSixlabdUealnaaBaaaleaacqWGebaraeqaaaGcbaWaaeWaaeaacqWGlbWsdaWgaaWcbaGaemiraqeabeaakiabgUcaRiabdIha4jabcIcaOiabdsha0jabcMcaPaGaayjkaiaawMcaamaaCaaaleqabaGaeGOmaidaaaaaaaaaaa@51EF@

Using eqs. (17) and (50), the calcium binding capacity becomes:

(52)κ=ymax⁡⋅KD(KD+x(t))2
 MathType@MTEF@5@5@+=feaafiart1ev1aaatCvAUfKttLearuWrP9MDH5MBPbIqV92AaeXatLxBI9gBaebbnrfifHhDYfgasaacH8akY=wiFfYdH8Gipec8Eeeu0xXdbba9frFj0=OqFfea0dXdd9vqai=hGuQ8kuc9pgc9s8qqaq=dirpe0xb9q8qiLsFr0=vr0=vr0dc8meaabaqaciaacaGaaeqabaqabeGadaaakeaafaqabeqacaaabaWaaeWaaeaacqaI1aqncqaIYaGmaiaawIcacaGLPaaaaeaaiiGacqWF6oWAcqGH9aqpdaWcaaqaaiabdMha5naaBaaaleaacyGGTbqBcqGGHbqycqGG4baEaeqaaOGaeyyXICTaem4saS0aaSbaaSqaaiabdseaebqabaaakeaadaqadaqaaiabdUealnaaBaaaleaacqWGebaraeqaaOGaey4kaSIaemiEaGNaeiikaGIaemiDaqNaeiykaKcacaGLOaGaayzkaaWaaWbaaSqabeaacqaIYaGmaaaaaaaaaaa@480E@

This, again, is identical to eq. (3) in [20].

In their study on Calcium diffusion, Naraghi and Neher [17] investigated a linearized mathematical model of diffusion and kinetics. For one buffer their results are contained in their eqs (AII.9) and (AII.10). This corresponds to our analysis when one sets the calcium source, our *α*(*t*), and our kinetic loss terms for free calcium, our -*γ x*(*t*), both to zero, accounting for the zero eigenvalues they report. Majewska et al. [19] focused on determining time constants for intracellular calcium kinetics experimentally. Their results show time scales in the range of 100 s of ms. This is the order of magnitude we have used in selecting our pump rate *γ *whose value we chose as 1/*γ *= 100 ms in our numerical simulations. Wagner and Keizer [16] again focused on diffusion of calcium. Their notation identifies a free calcium concentration [Ca^2+^], which is just our *x*(*t*), and a concentration of calcium bound to a mobile buffer [CaB_m_], which is precisely our y(t), and finally the concentration of the mobile buffer itself [B_m_], which is our y_max_-y(t). They do not have source terms for the calcium influx, our *α*(*t*), or kinetic loss terms for free calcium, our -*γ x*(*t*). Ignoring diffusion and these sources and sinks of [Ca^2+^], their eqs. (2), (3), and (4), are precisely our eqs. (1) and (2) above. It is important they do not analyze the nonlinear ordinary (kinetic) differential equations that result when diffusion is not important. Using the estimates of Zhou and Neher for the diffusion constants to be about 300 *μ*m^2^/s this translates to a time for diffusion over a cellular scale to be about 3 ms which is much shorter than the kinetic time constants we consider or are discussed by Majewska et al. [19]. This gives our rationale for ignoring diffusion and focusing on properties of the nonlinear kinetics.

### Appendix I: perturbation analysis of the nonlinear solution

We begin by making eqs. (1) and (2) dimensionless. There are three quantities with the dimensions of (time)^-1^: k_b, *γ*_, and k_f_y_max_. We express our indicator kinetic equations in terms of the two dimensionless variables which can be made from these

(A1)     *R*_*b *_= *k*_*b*_/*γ*;     *R*_*f *_= *k*_*f *_*y*_max_/*γ*.

We also scale *x*(*t*) and *y*(*t*) with the initial indicator concentration y_max _and the time by the pump rate *γ*:

(A2)     *x*(*t*) → *y*_max _*X*(*t*);     *y*(*t*) → *y*_max _*Y*(*t*);     *t *→ *t*/*γ*;

Thus, in these new dimensionless variables, free calcium and calcium-bound indicator concentrations are given as fractions of the initial free indicator concentration, and the forward and backward rates are given relative to the pump rate.

The kinetic eqs. (1) and (2) now become:

(A3)     *X'*(*t*) = *α*(*t*)/(*γ*·*y*_max_) - *X*(*t*) - *Y'*(*t*)

(A4)     *Y'*(*t*) = *R*_*f *_*X*(*t*)·(1 - *Y*(*t*)) - *R*_*b *_*Y*(*t*).

We chose as an approximate solution of these equations functions

(A5)Y0(t)=RfX0(t)Rb+RfX0(t),
 MathType@MTEF@5@5@+=feaafiart1ev1aaatCvAUfKttLearuWrP9MDH5MBPbIqV92AaeXatLxBI9gBaebbnrfifHhDYfgasaacH8akY=wiFfYdH8Gipec8Eeeu0xXdbba9frFj0=OqFfea0dXdd9vqai=hGuQ8kuc9pgc9s8qqaq=dirpe0xb9q8qiLsFr0=vr0=vr0dc8meaabaqaciaacaGaaeqabaqabeGadaaakeaafaqabeqacaaabaWaaeWaaeaacqqGbbqqcqaI1aqnaiaawIcacaGLPaaaaeaacqWGzbqwdaWgaaWcbaGaeGimaadabeaakiabcIcaOiabdsha0jabcMcaPiabg2da9maalaaabaGaemOuai1aaSbaaSqaaiabdAgaMbqabaGccqWGybawdaWgaaWcbaGaeGimaadabeaakiabcIcaOiabdsha0jabcMcaPaqaaiabdkfasnaaBaaaleaacqWGIbGyaeqaaOGaey4kaSIaemOuai1aaSbaaSqaaiabdAgaMbqabaGccqWGybawdaWgaaWcbaGaeGimaadabeaakiabcIcaOiabdsha0jabcMcaPaaacqGGSaalaaaaaa@4BBD@

for which

(A6)Y′0(t)=RfRb[Rb+RfX0(t)]2⋅X′0(t).
 MathType@MTEF@5@5@+=feaafiart1ev1aaatCvAUfKttLearuWrP9MDH5MBPbIqV92AaeXatLxBI9gBaebbnrfifHhDYfgasaacH8akY=wiFfYdH8Gipec8Eeeu0xXdbba9frFj0=OqFfea0dXdd9vqai=hGuQ8kuc9pgc9s8qqaq=dirpe0xb9q8qiLsFr0=vr0=vr0dc8meaabaqaciaacaGaaeqabaqabeGadaaakeaafaqabeqacaaabaWaaeWaaeaacqqGbbqqcqaI2aGnaiaawIcacaGLPaaaaeaacuWGzbqwgaqbamaaBaaaleaacqaIWaamaeqaaOGaeiikaGIaemiDaqNaeiykaKIaeyypa0ZaaSaaaeaacqWGsbGudaWgaaWcbaGaemOzaygabeaakiabdkfasnaaBaaaleaacqWGIbGyaeqaaaGcbaWaamWaaeaacqWGsbGudaWgaaWcbaGaemOyaigabeaakiabgUcaRiabdkfasnaaBaaaleaacqWGMbGzaeqaaOGaemiwaG1aaSbaaSqaaiabicdaWaqabaGccqGGOaakcqWG0baDcqGGPaqkaiaawUfacaGLDbaadaahaaWcbeqaaiabikdaYaaaaaGccqGHflY1cuWGybawgaqbamaaBaaaleaacqaIWaamaeqaaOGaeiikaGIaemiDaqNaeiykaKIaeiOla4caaaaa@53F0@

This solution is suggested by the vanishing of the right hand side of eq. (A4) as well as by the fixed point solution, true when *X*(*t*) is time independent. Another motivation for this approximate solution is that when both R_b _and R_f _are large, the right hand side of eq. (A4) would make the rate of change of the calcium bound indicator vary quite rapidly unless the balance indicated by eq. (A5) were maintained.

To determine when this solution is accurate, we make perturbations

(A7)     *X*(*t*) = *X*_0_(*t*) + Δ_*X*_(*t*)

(A8)     *Y*(*t*) = *Y*_0_(*t*) + Δ_*Y*_(*t*),

and linearize the equations in Δ_*X*_(*t*) and Δ_*Y*_(*t*). From eqs. (A3) and (A4) we obtain, to first order in the perturbations,

(A9)dΔX(t)dt=−ΔX(t)[1+RbRfη]+ηΔY(t)
 MathType@MTEF@5@5@+=feaafiart1ev1aaatCvAUfKttLearuWrP9MDH5MBPbIqV92AaeXatLxBI9gBaebbnrfifHhDYfgasaacH8akY=wiFfYdH8Gipec8Eeeu0xXdbba9frFj0=OqFfea0dXdd9vqai=hGuQ8kuc9pgc9s8qqaq=dirpe0xb9q8qiLsFr0=vr0=vr0dc8meaabaqaciaacaGaaeqabaqabeGadaaakeaafaqabeqacaaabaWaaeWaaeaacqqGbbqqcqaI5aqoaiaawIcacaGLPaaaaeaadaWcaaqaaiabdsgaKjabfs5aenaaBaaaleaacqWGybawaeqaaOGaeiikaGIaemiDaqNaeiykaKcabaGaemizaqMaemiDaqhaaiabg2da9iabgkHiTiabfs5aenaaBaaaleaacqWGybawaeqaaOGaeiikaGIaemiDaqNaeiykaKYaamWaaeaacqaIXaqmcqGHRaWkdaWcaaqaaiabdkfasnaaBaaaleaacqWGIbGyaeqaaOGaemOuai1aaSbaaSqaaiabdAgaMbqabaaakeaaiiGacqWF3oaAaaaacaGLBbGaayzxaaGaey4kaSIae83TdGMaeuiLdq0aaSbaaSqaaiabdMfazbqabaGccqGGOaakcqWG0baDcqGGPaqkaaaaaa@55C3@

(A10)dΔY(t)dt=ΔX(t)RfRbη−ηΔY(t)−dY0(t)dt,
 MathType@MTEF@5@5@+=feaafiart1ev1aaatCvAUfKttLearuWrP9MDH5MBPbIqV92AaeXatLxBI9gBaebbnrfifHhDYfgasaacH8akY=wiFfYdH8Gipec8Eeeu0xXdbba9frFj0=OqFfea0dXdd9vqai=hGuQ8kuc9pgc9s8qqaq=dirpe0xb9q8qiLsFr0=vr0=vr0dc8meaabaqaciaacaGaaeqabaqabeGadaaakeaafaqabeqacaaabaWaaeWaaeaacqqGbbqqcqaIXaqmcqaIWaamaiaawIcacaGLPaaaaeaadaWcaaqaaiabdsgaKjabfs5aenaaBaaaleaacqWGzbqwaeqaaOGaeiikaGIaemiDaqNaeiykaKcabaGaemizaqMaemiDaqhaaiabg2da9iabfs5aenaaBaaaleaacqWGybawaeqaaOGaeiikaGIaemiDaqNaeiykaKYaaSaaaeaacqWGsbGudaWgaaWcbaGaemOzaygabeaakiabdkfasnaaBaaaleaacqWGIbGyaeqaaaGcbaacciGae83TdGgaaiabgkHiTiab=D7aOjabfs5aenaaBaaaleaacqWGzbqwaeqaaOGaeiikaGIaemiDaqNaeiykaKIaeyOeI0YaaSaaaeaacqWGKbazcqWGzbqwdaWgaaWcbaGaeGimaadabeaakiabcIcaOiabdsha0jabcMcaPaqaaiabdsgaKjabdsha0baacqGGSaalaaaaaa@5D6F@

where

(A11)     *η *= *R*_*b *_+ *R*_*f *_*X*_0_(*t*),

and *X*_0_(*t*) satisfies

(A12)X′0(t)=α(t)γ⋅ymax⁡−X0(t).
 MathType@MTEF@5@5@+=feaafiart1ev1aaatCvAUfKttLearuWrP9MDH5MBPbIqV92AaeXatLxBI9gBaebbnrfifHhDYfgasaacH8akY=wiFfYdH8Gipec8Eeeu0xXdbba9frFj0=OqFfea0dXdd9vqai=hGuQ8kuc9pgc9s8qqaq=dirpe0xb9q8qiLsFr0=vr0=vr0dc8meaabaqaciaacaGaaeqabaqabeGadaaakeaafaqabeqacaaabaWaaeWaaeaacqqGbbqqcqaIXaqmcqaIYaGmaiaawIcacaGLPaaaaeaacuWGybawgaqbamaaBaaaleaacqaIWaamaeqaaOGaeiikaGIaemiDaqNaeiykaKIaeyypa0ZaaSaaaeaaiiGacqWFXoqycqGGOaakcqWG0baDcqGGPaqkaeaacqWFZoWzcqGHflY1cqWG5bqEdaWgaaWcbaGagiyBa0MaeiyyaeMaeiiEaGhabeaaaaGccqGHsislcqWGybawdaWgaaWcbaGaeGimaadabeaakiabcIcaOiabdsha0jabcMcaPiabc6caUaaaaaa@4DAC@

These are very similar to those for the linearized problem discussed in the text. The key differences are that *η *= *R*_*b *_+ *R*_*f *_*X*_0_(*t*) is time dependent and there is an inhomogeneous term in the equations for Δ_*Y*_(*t*). Since the solution for the unperturbed *X*_0_(*t*) is a low pass filtered version of the calcium influx, we take it as a positive constant, slowly varying, in the perturbation equations.

The equation for Δ(*t*) = (Δ_*X*_(*t*), Δ_*Y*_(*t*)) written in matrix form is

(A13)dΔ(t)dt=MΔ(t)+F(t),
 MathType@MTEF@5@5@+=feaafiart1ev1aaatCvAUfKttLearuWrP9MDH5MBPbIqV92AaeXatLxBI9gBaebbnrfifHhDYfgasaacH8akY=wiFfYdH8Gipec8Eeeu0xXdbba9frFj0=OqFfea0dXdd9vqai=hGuQ8kuc9pgc9s8qqaq=dirpe0xb9q8qiLsFr0=vr0=vr0dc8meaabaqaciaacaGaaeqabaqabeGadaaakeaafaqabeqacaaabaWaaeWaaeaacqqGbbqqcqaIXaqmcqaIZaWmaiaawIcacaGLPaaaaeaadaWcaaqaaiabdsgaKjabfs5aejabcIcaOiabdsha0jabcMcaPaqaaiabdsgaKjabdsha0baacqGH9aqpcqWGnbqtcqqHuoarcqGGOaakcqWG0baDcqGGPaqkcqGHRaWkcqWGgbGrcqGGOaakcqWG0baDcqGGPaqkcqGGSaalaaaaaa@4687@

with F(t)=(0,−dY0(t)dt)
 MathType@MTEF@5@5@+=feaafiart1ev1aaatCvAUfKttLearuWrP9MDH5MBPbIqV92AaeXatLxBI9gBaebbnrfifHhDYfgasaacH8akY=wiFfYdH8Gipec8Eeeu0xXdbba9frFj0=OqFfea0dXdd9vqai=hGuQ8kuc9pgc9s8qqaq=dirpe0xb9q8qiLsFr0=vr0=vr0dc8meaabaqaciaacaGaaeqabaqabeGadaaakeaacqWGgbGrcqGGOaakcqWG0baDcqGGPaqkcqGH9aqpcqGGOaakcqaIWaamcqGGSaalcqGHsisldaWcaaqaaiabdsgaKjabdMfaznaaBaaaleaacqaIWaamaeqaaOGaeiikaGIaemiDaqNaeiykaKcabaGaemizaqMaemiDaqhaaiabcMcaPaaa@3FFC@. The eigenvalues of the matrix *M *are

(A14)     *λ*_1,2 _= -*C *± *D*

**Figure 4:**
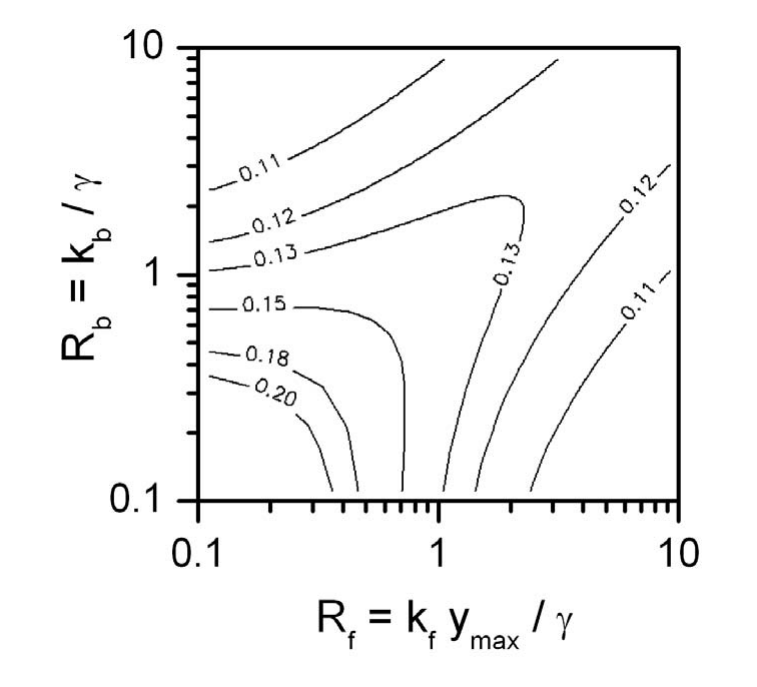
Relaxation time-constant (eq. (A14) with X_0 _= 1.0) as a function of the two dimensionless kinetic parameters Rb and Rf. Numbers on the iso-*τ *contour lines indicate the value in seconds. Compare with Fig. 2d.

With C=12[1+η+RbRfη]
 MathType@MTEF@5@5@+=feaafiart1ev1aaatCvAUfKttLearuWrP9MDH5MBPbIqV92AaeXatLxBI9gBaebbnrfifHhDYfgasaacH8akY=wiFfYdH8Gipec8Eeeu0xXdbba9frFj0=OqFfea0dXdd9vqai=hGuQ8kuc9pgc9s8qqaq=dirpe0xb9q8qiLsFr0=vr0=vr0dc8meaabaqaciaacaGaaeqabaqabeGadaaakeaacqWGdbWqcqGH9aqpdaWcaaqaaiabigdaXaqaaiabikdaYaaadaWadaqaaiabigdaXiabgUcaRGGaciab=D7aOjabgUcaRmaalaaabaGaemOuai1aaSbaaSqaaiabdkgaIbqabaGccqWGsbGudaWgaaWcbaGaemOzaygabeaaaOqaaiab=D7aObaaaiaawUfacaGLDbaaaaa@3E2B@

and D=12[1+η+RbRfη]2−4η=12[1−η+RbRfη]2+4RbRf,
 MathType@MTEF@5@5@+=feaafiart1ev1aaatCvAUfKttLearuWrP9MDH5MBPbIqV92AaeXatLxBI9gBaebbnrfifHhDYfgasaacH8akY=wiFfYdH8Gipec8Eeeu0xXdbba9frFj0=OqFfea0dXdd9vqai=hGuQ8kuc9pgc9s8qqaq=dirpe0xb9q8qiLsFr0=vr0=vr0dc8meaabaqaciaacaGaaeqabaqabeGadaaakeaacqWGebarcqGH9aqpdaWcaaqaaiabigdaXaqaaiabikdaYaaadaGcaaqaamaadmaabaGaeGymaeJaey4kaSccciGae83TdGMaey4kaSYaaSaaaeaacqWGsbGudaWgaaWcbaGaemOyaigabeaakiabdkfasnaaBaaaleaacqWGMbGzaeqaaaGcbaGaeq4TdGgaaaGaay5waiaaw2faamaaCaaaleqabaGaeGOmaidaaOGaeyOeI0IaeGinaqJae83TdGgaleqaaOGaeyypa0ZaaSaaaeaacqaIXaqmaeaacqaIYaGmaaWaaOaaaeaadaWadaqaaiabigdaXiabgkHiTiab=D7aOjabgUcaRmaalaaabaGaemOuai1aaSbaaSqaaiabdkgaIbqabaGccqWGsbGudaWgaaWcbaGaemOzaygabeaaaOqaaiab=D7aObaaaiaawUfacaGLDbaadaahaaWcbeqaaiabikdaYaaakiabgUcaRiabisda0iabdkfasnaaBaaaleaacqWGIbGyaeqaaOGaemOuai1aaSbaaSqaaiabdAgaMbqabaaabeaakiabcYcaSaaa@5CD2@,

so *C, D *>0 and *D*<*C*, so both eigenvalues are negative. This means the solution is trying to drive Δ(*t*) = (Δ_*X*_(*t*), Δ_*Y*_(*t*)) to zero, with "bumps" from the forcing term. If the forcing term is bounded above, that is the derivative (A6) remains below some maximum value while the calcium current is flowing, the solutions Δ(*t*) = (Δ_*X*_(*t*), Δ_*Y*_(*t*)) go to zero. In these weak conditions, the assumed solutions (*X*_0_(*t*),*Y*_0_(*t*)) are stable. The larger time-constant is shown in Fig. 4 as a function of R_b _and R_f_. It agrees with the rms values shown in Fig. 2d.

### Appendix II: relating the fluorescence signal to calcium-bound indicator concentration

From eqs. (19) and (32), it is important to note that *α*(t) does not scale with y(t). Therefore, the indicator concentration enters these equations as an absolute concentration. Otherwise, the calculated time-course of the calcium influx will be incorrect (and not just by a factor!). Usually, however, the indicator concentration is not available directly, but rather as fluorescence values, in most cases as ΔF/F, i.e. fluorescence changes relative to a reference fluorescence F(0) obtained just before the start of an experiment. The fluorescence value F(t) is the sum of the fluorescence of the indicator with bound calcium. i.e. y(t), and free indicator concentrations, i.e. z_0_-y(t), each one contributing to the total fluorescence by a factor f_b _(bound) and f_f _(free), respectively:

(A15)     *F*(*t*) = *f*_*b*_·*y*(*t*) + *f*_*f*_·(*y*_max _- *y*(*t*))

The factors f_b _and f_f _both can be determined experimentally from the fluorescence of a calcium-free and a calcium-saturated indicator solution. Using the maximum fluorescence change (ΔF/F)_max _of the indicator as (f_b _- f_f_)/f_f_, the following relation then holds between y(t) and ΔF/F:

(A16)ΔFF(t)=F(t)−F(0)F(0)=[y(t)−y(0)]⋅(ΔF/F)max⁡y(0)⋅(ΔF/F)max⁡+ymax⁡
 MathType@MTEF@5@5@+=feaafiart1ev1aaatCvAUfKttLearuWrP9MDH5MBPbIqV92AaeXatLxBI9gBaebbnrfifHhDYfgasaacH8akY=wiFfYdH8Gipec8Eeeu0xXdbba9frFj0=OqFfea0dXdd9vqai=hGuQ8kuc9pgc9s8qqaq=dirpe0xb9q8qiLsFr0=vr0=vr0dc8meaabaqaciaacaGaaeqabaqabeGadaaakeaafaqabeqacaaabaWaaeWaaeaacqqGbbqqcqaIXaqmcqaI2aGnaiaawIcacaGLPaaaaeaadaWcaaqaaiabfs5aejabdAeagbqaaiabdAeagbaacqGGOaakcqWG0baDcqGGPaqkcqGH9aqpdaWcaaqaaiabdAeagjabcIcaOiabdsha0jabcMcaPiabgkHiTiabdAeagjabcIcaOiabicdaWiabcMcaPaqaaiabdAeagjabcIcaOiabicdaWiabcMcaPaaacqGH9aqpdaWcaaqaamaadmaabaGaemyEaKNaeiikaGIaemiDaqNaeiykaKIaeyOeI0IaemyEaKNaeiikaGIaeGimaaJaeiykaKcacaGLBbGaayzxaaGaeyyXIC9aaeWaaeaacqqHuoarcqWGgbGrcqGGVaWlcqWGgbGraiaawIcacaGLPaaadaWgaaWcbaGagiyBa0MaeiyyaeMaeiiEaGhabeaaaOqaaiabdMha5jabcIcaOiabicdaWiabcMcaPiabgwSixpaabmaabaGaeuiLdqKaemOrayKaei4la8IaemOrayeacaGLOaGaayzkaaWaaSbaaSqaaiGbc2gaTjabcggaHjabcIha4bqabaGccqGHRaWkcqWG5bqEdaWgaaWcbaGagiyBa0MaeiyyaeMaeiiEaGhabeaaaaaaaaaa@765F@

Solving eq (A16) for y(t) yields:

(A17)y(t)=ΔFF(t)⋅(y(0)+ymax⁡(ΔF/Fmax⁡))+y(0).
 MathType@MTEF@5@5@+=feaafiart1ev1aaatCvAUfKttLearuWrP9MDH5MBPbIqV92AaeXatLxBI9gBaebbnrfifHhDYfgasaacH8akY=wiFfYdH8Gipec8Eeeu0xXdbba9frFj0=OqFfea0dXdd9vqai=hGuQ8kuc9pgc9s8qqaq=dirpe0xb9q8qiLsFr0=vr0=vr0dc8meaabaqaciaacaGaaeqabaqabeGadaaakeaafaqabeqacaaabaWaaeWaaeaacqqGbbqqcqaIXaqmcqaI3aWnaiaawIcacaGLPaaaaeaacqWG5bqEcqGGOaakcqWG0baDcqGGPaqkcqGH9aqpdaWcaaqaaiabfs5aejabdAeagbqaaiabdAeagbaacqGGOaakcqWG0baDcqGGPaqkcqGHflY1daqadaqaaiabdMha5jabcIcaOiabicdaWiabcMcaPiabgUcaRmaalaaabaGaemyEaK3aaSbaaSqaaiGbc2gaTjabcggaHjabcIha4bqabaaakeaadaqadaqaaiabfs5aejabdAeagjabc+caViabdAeagnaaBaaaleaacyGGTbqBcqGGHbqycqGG4baEaeqaaaGccaGLOaGaayzkaaaaaaGaayjkaiaawMcaaiabgUcaRiabdMha5jabcIcaOiabicdaWiabcMcaPiabc6caUaaaaaa@5C91@

When using indicators based on fluorescence resonance energy transfer ('FRET'), results are usually expressed in the relative change of the fluorescence ratio obtained at two different wavelengths, one from the donor fluorophore F_1_, and the other from the acceptor fluorophore F_2_, respectively:

(A18)ΔRR=F1(t)/F2(t)−F1(0)/F2(0)F1(0)/F2(0)=F1(t)F2(0)F2(t)F1(0)−1
 MathType@MTEF@5@5@+=feaafiart1ev1aaatCvAUfKttLearuWrP9MDH5MBPbIqV92AaeXatLxBI9gBaebbnrfifHhDYfgasaacH8akY=wiFfYdH8Gipec8Eeeu0xXdbba9frFj0=OqFfea0dXdd9vqai=hGuQ8kuc9pgc9s8qqaq=dirpe0xb9q8qiLsFr0=vr0=vr0dc8meaabaqaciaacaGaaeqabaqabeGadaaakeaafaqabeqacaaabaWaaeWaaeaacqqGbbqqcqaIXaqmcqaI4aaoaiaawIcacaGLPaaaaeaadaWcaaqaaiabfs5aejabdkfasbqaaiabdkfasbaacqGH9aqpdaWcaaqaaiabdAeagnaaBaaaleaacqaIXaqmaeqaaOGaeiikaGIaemiDaqNaeiykaKIaei4la8IaemOray0aaSbaaSqaaiabikdaYaqabaGccqGGOaakcqWG0baDcqGGPaqkcqGHsislcqWGgbGrdaWgaaWcbaGaeGymaedabeaakiabcIcaOiabicdaWiabcMcaPiabc+caViabdAeagnaaBaaaleaacqaIYaGmaeqaaOGaeiikaGIaeGimaaJaeiykaKcabaGaemOray0aaSbaaSqaaiabigdaXaqabaGccqGGOaakcqaIWaamcqGGPaqkcqGGVaWlcqWGgbGrdaWgaaWcbaGaeGOmaidabeaakiabcIcaOiabicdaWiabcMcaPaaacqGH9aqpdaWcaaqaaiabdAeagnaaBaaaleaacqaIXaqmaeqaaOGaeiikaGIaemiDaqNaeiykaKIaemOray0aaSbaaSqaaiabikdaYaqabaGccqGGOaakcqaIWaamcqGGPaqkaeaacqWGgbGrdaWgaaWcbaGaeGOmaidabeaakiabcIcaOiabdsha0jabcMcaPiabdAeagnaaBaaaleaacqaIXaqmaeqaaOGaeiikaGIaeGimaaJaeiykaKcaaiabgkHiTiabigdaXaaaaaa@6F55@

Inserting eq. (A15) for each wavelength, the relation to the indicator concentration becomes:

(A19)ΔRR=ymax⁡⋅(y(t)−y(0))⋅(fb1ff2−fb2ff1)[y(t)⋅(fb2−ff2)+ymax⁡ff2]⋅[y(t)⋅(fb1−ff1)+ymax⁡ff1]
 MathType@MTEF@5@5@+=feaafiart1ev1aaatCvAUfKttLearuWrP9MDH5MBPbIqV92AaeXatLxBI9gBaebbnrfifHhDYfgasaacH8akY=wiFfYdH8Gipec8Eeeu0xXdbba9frFj0=OqFfea0dXdd9vqai=hGuQ8kuc9pgc9s8qqaq=dirpe0xb9q8qiLsFr0=vr0=vr0dc8meaabaqaciaacaGaaeqabaqabeGadaaakeaafaqabeqacaaabaWaaeWaaeaacqqGbbqqcqaIXaqmcqaI5aqoaiaawIcacaGLPaaaaeaadaWcaaqaaiabfs5aejabdkfasbqaaiabdkfasbaacqGH9aqpdaWcaaqaaiabdMha5naaBaaaleaacyGGTbqBcqGGHbqycqGG4baEaeqaaOGaeyyXIC9aaeWaaeaacqWG5bqEcqGGOaakcqWG0baDcqGGPaqkcqGHsislcqWG5bqEcqGGOaakcqaIWaamcqGGPaqkaiaawIcacaGLPaaacqGHflY1daqadaqaaiabdAgaMnaaBaaaleaacqWGIbGycqaIXaqmaeqaaOGaemOzay2aaSbaaSqaaiabdAgaMjabikdaYaqabaGccqGHsislcqWGMbGzdaWgaaWcbaGaemOyaiMaeGOmaidabeaakiabdAgaMnaaBaaaleaacqWGMbGzcqaIXaqmaeqaaaGccaGLOaGaayzkaaaabaWaamWaaeaacqWG5bqEcqGGOaakcqWG0baDcqGGPaqkcqGHflY1daqadaqaaiabdAgaMnaaBaaaleaacqWGIbGycqaIYaGmaeqaaOGaeyOeI0IaemOzay2aaSbaaSqaaiabdAgaMjabikdaYaqabaaakiaawIcacaGLPaaacqGHRaWkcqWG5bqEdaWgaaWcbaGagiyBa0MaeiyyaeMaeiiEaGhabeaakiabdAgaMnaaBaaaleaacqWGMbGzcqaIYaGmaeqaaaGccaGLBbGaayzxaaGaeyyXIC9aamWaaeaacqWG5bqEcqGGOaakcqWG0baDcqGGPaqkcqGHflY1daqadaqaaiabdAgaMnaaBaaaleaacqWGIbGycqaIXaqmaeqaaOGaeyOeI0IaemOzay2aaSbaaSqaaiabdAgaMjabigdaXaqabaaakiaawIcacaGLPaaacqGHRaWkcqWG5bqEdaWgaaWcbaGagiyBa0MaeiyyaeMaeiiEaGhabeaakiabdAgaMnaaBaaaleaacqWGMbGzcqaIXaqmaeqaaaGccaGLBbGaayzxaaaaaaaaaaa@9A97@
